# Benchmarking sequencing methods and tools that facilitate the study of alternative polyadenylation

**DOI:** 10.1186/s13059-021-02502-z

**Published:** 2021-10-14

**Authors:** Ankeeta Shah, Briana E. Mittleman, Yoav Gilad, Yang I. Li

**Affiliations:** 1 Genetics, Genomics, and Systems Biology, University of Chicago, Chicago, IL USA; 2 Section of Genetic Medicine, Department of Medicine, University of Chicago, Chicago, IL USA; 3 Department of Human Genetics, University of Chicago, Chicago, IL USA

**Keywords:** Benchmarking, Alternative polyadenylation, RNA processing, Isoform analysis, RNA-seq, 3′-Seq, PacBio Iso-Seq, Long-read sequencing, QTL

## Abstract

**Background:**

Alternative cleavage and polyadenylation (APA), an RNA processing event, occurs in over 70% of human protein-coding genes. APA results in mRNA transcripts with distinct 3′ ends. Most APA occurs within 3′ UTRs, which harbor regulatory elements that can impact mRNA stability, translation, and localization.

**Results:**

APA can be profiled using a number of established computational tools that infer polyadenylation sites from standard, short-read RNA-seq datasets. Here, we benchmarked a number of such tools—TAPAS, QAPA, DaPars2, GETUTR, and APATrap— against 3′-Seq, a specialized RNA-seq protocol that enriches for reads at the 3′ ends of genes, and Iso-Seq, a Pacific Biosciences (PacBio) single-molecule full-length RNA-seq method in their ability to identify polyadenylation sites and quantify polyadenylation site usage. We demonstrate that 3′-Seq and Iso-Seq are able to identify and quantify the usage of polyadenylation sites more reliably than computational tools that take short-read RNA-seq as input. However, we find that running one such tool, QAPA, with a set of polyadenylation site annotations derived from small quantities of 3′-Seq or Iso-Seq can reliably quantify variation in APA across conditions, such asacross genotypes, as demonstrated by the successful mapping of alternative polyadenylation quantitative trait loci (apaQTL).

**Conclusions:**

We envisage that our analyses will shed light on the advantages of studying APA with more specialized sequencing protocols, such as 3′-Seq or Iso-Seq, and the limitations of studying APA with short-read RNA-seq. We provide a computational pipeline to aid in the identification of polyadenylation sites and quantification of polyadenylation site usages using Iso-Seq data as input.

**Supplementary Information:**

The online version contains supplementary material available at 10.1186/s13059-021-02502-z.

## Background

Although the human genome only harbors about twenty thousand protein-coding genes, the human transcriptome encodes ten times that number, or two hundred thousand unique transcripts [1]. Over the last two decades, it has become increasingly apparent that RNA processing events, such as alternative splicing, alternative transcription start site usage, and alternative polyadenylation, are drivers of the human transcriptome’s astonishing complexity, allowing single genes to encode a repertoire of transcript isoforms [2]. Alternative polyadenylation (APA), the process by which a single gene is able to produce multiple mRNA isoforms with distinct 3′ ends, is a critical RNA processing event that can affect the stability, localization, transport, and translation of mRNA [3–8].

The cleavage and polyadenylation reaction is controlled by sequence elements upstream and downstream of the cleavage and polyadenylation signal (PAS) site. These sequence elements are recognized by several proteins and protein complexes that are recruited upon transcription of the PAS site by RNA polymerase II. Coordinated recognition of the signal site, a hexameric A[A/U]UAAA sequence or variant thereof, ~20–30 nucleotides upstream of the PAS site [9, 10], and a GU-rich downstream sequence element, ~10–30 nucleotides downstream of the PAS site [8, 11, 12], is mediated by the cleavage and polyadenylation specificity factor (CPSF) and cleavage stimulation factor (CstF) complexes. In addition to harboring alternative splice sites, genes may also harbor alternative PAS sites. By using different PAS sites, mRNA transcripts are produced with different 3′ UTR lengths, for example, which can contain distinct cis-regulatory elements, such as miRNA binding sites or RNA binding protein sites [13]. This process, APA, can be important in the regulation of normal differentiation and development [14, 15] or mis-regulation in the context of disease [16].

The rise of next-generation, high-throughput RNA sequencing (RNA-seq) [17] has allowed researchers to, without a priori knowledge of gene annotations, quantitatively measure gene expression, discover novel transcripts, and measure the relative abundance of distinct transcript isoforms. Studies using standard RNA-seq estimate that ~70% of genes in the human genome harbor multiple PAS sites, most of which are localized within 3′ UTRs [18]. To study the effect of APA on gene regulation, a number of research groups have developed computational tools that leverage standard RNA-seq data to identify PAS sites and quantify polyadenylation site usages (PAUs). The growing number of such tools is a result of the extensive availability of short-read RNA-seq data across conditions, including across cell types, individuals, and organisms [19–21].

**Fig. 1:**
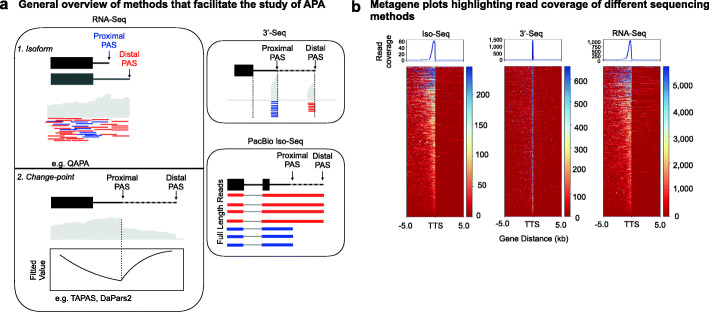
Overview of three sequencing methods utilized to study APA. **a** Schematic of sequencing methods—RNA-Seq, 3′-Seq, and Iso-Seq—that facilitate the study of APA. Examples of RNA-Seq methods to study APA. **b** Metagene plots showing read coverage centered around the transcription termination site (TTS) for five RNA-Seq libraries, five 3′-Seq libraries, and eight Iso-Seq libraries collected from YRI LCLs

Some existing approaches that leverage short-read RNA-seq to study APA rely on estimating PAU based on transcript-level abundance [19, 22] (Fig. 1a). For example, QAPA calculates the relative proportion of every gene isoform using a combination of existing tools, namely Sailfish [23] and Salmon [24], and PAS site annotations [19]. The use of annotations may, at times, be a drawback as certain PAS site databases might currently be missing annotation information in particular cell types or organisms of interest, biasing analyses comparing PAU across conditions. Other methods perform de novo identification of PAS sites by using a change-point model, which is based on a generalized likelihood ratio statistic of identifying transcript length changes [20, 21, 25] (Fig. 1a). For example, DaPars2 infers the location of a single proximal PAS site within 3′ UTRs [20, 25, 26]. GETUTR identifies multiple PAS sites within 3′ UTRs using kernel density estimation [27]. APATrap identifies multiple PAS sites within novel 3′ UTRs and 3′ UTR extensions using a mean squared error model [28]. Finally, TAPAS infers PAS sites within and upstream of 3′ UTRs [21].

While these methods have provided valuable insights into the landscape of APA across a myriad of biological contexts, there are a number of challenges associated with studying APA with short-read RNA-seq. Generally, the estimation of isoform abundance from short-read RNA-seq is statistically challenging because short-read protocols tend to sample small portions of transcripts, and alternative transcripts often have substantial overlap [29]. Specific biases exist due to the fact that standard RNA-seq protocols include multiple PCR amplification steps during library preparation [30]. There is also bias in sequencing repetitive regions of the genome [31] and the issue of some short reads not aligning uniquely to a reference genome of interest [32]. Most importantly, the coverage of RNA-seq at the 3′ end of mRNA transcripts is often limited, which makes estimation of the PAU particularly difficult. 3′ end sequencing (3′-Seq), which enriches for reads covering the 3′ end of genes [33], overcomes the issue of limited coverage but suffers from some of the other biases associated with standard RNA-seq, such as mapping errors associated with reads derived from repetitive regions in the genome (Fig. 1b).

Because of the biases and analytical challenges associated with short-read sequencing protocols and their variants, we took advantage of the Pacific Biosciences (PacBio) single-molecule isoform-sequencing (Iso-Seq) [34] to more precisely identify PAS sites and quantify PAUs. We reasoned that because Iso-Seq enables sequencing through polyA tails [34], some, but not all, of the biases associated with studying APA using 3′-Seq and short-read RNA-seq data would be minimized. Supporting this view, a recent study surveyed the sorghum transcriptome using single-molecule long reads, allowing for enhanced sorghum gene isoform annotation without the need for transcript reconstruction [35]. In this study, we benchmarked the ability to study APA on a genome-wide scale in humans using short-read RNA-seq-based computational tools—TAPAS [21], DaPars2 [20, 25, 26], QAPA [19], GETUTR [27], and APATrap [28], against 3′-Seq and PacBio Iso-Seq. While there are many computational tools available that allow one to study APA, we chose these tools specifically because they leverage distinct approaches for studying APA (Fig. 1a). We highlight some of the relative advantages and disadvantages of these sequencing methods and tools to inform the scientific community about what might best serve study goals.

## Results

### Identification and quantification of PAS sites using PacBio Iso-Seq

**Fig. 2:**
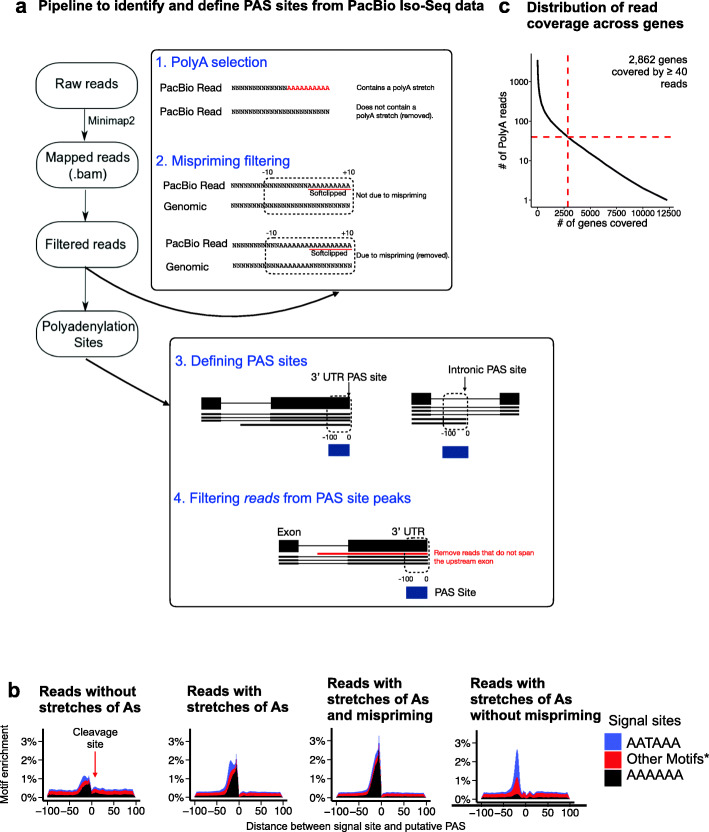
Profiling APA with Iso-Seq. **a** Workflow for identifying PAS sites using Iso-Seq. First, circular consensus reads were mapped to a reference genome. Next, Iso-Seq reads went through a series of two refinement steps to select for reads that contain stretches of As that are not due mispriming, resulting in a final set of reads that contain polyA tails. **b** Meta-gene barplots showing enrichment of signal site motifs ~20–30 nucleotides upstream of the putative cleavage site within these filtered reads. There are six consensus signal site motifs, with AATAAA being the most common. Other signal sites represented as "Other Motifs" include ATTAAA, AGTAAA, TATAAA, CATAAA, and GATAAA. AAAAAA served as a negative control. **c** We were able to accurately identify PAS sites and quantify PAUs for 2862 genes, which have an Iso-Seq coverage of ≥ 40 reads

To define a set of PAS sites to benchmark against, we compiled eight polyA-selected PacBio Iso-Seq lymphoblastoid cell line (LCL) samples. Specifically, we generated five libraries for Yoruba (YRI) LCLs GM18501, GM18504, GM19144, GM19239, and GM19153 [36] and obtained three previously published Central European (CEU) LCL libraries for GM12878, GM12891, and GM12892 [36, 37]. High quality consensus circular sequences (CCS) from each of the eight libraries were mapped to the hg19 human reference genome using minimap2 (v2.2.15) [38] separately (Fig. 2a). In order to maximize power to subsequently identify PAS sites, aligned reads from the eight libraries were pooled together, resulting in a total of 2.83 million reads that were used in all downstream analyses (the “Methods” section).

Aligned reads containing polyA tails were extracted from the alignment files after performing a series of filtering steps, including filtering based on the length and adenosine composition of polyA tails and filtering out internal priming or mispriming to minimize false-positive PAS site identification (Fig. 2a). In brief, reads that contained a stretch of at least six adenosines were retained. Moreover, because Iso-Seq reads should contain polyA tails that are not encoded in genomic DNA, we scanned the 10 nucleotides flanking either side of putative cleavage site in the genome for a stretch of at least six adenosines. If the genome did indeed contain a stretch of adenosines, we subsequently filtered out the associated reads for potential mispriming (the “Methods” section).

After filtering, we were left with 1.58 million reads with polyA tails and likely not misprimed. Using these 1.58 million reads, PAS sites were individually defined as the 100 nucleotide window between the putative cleavage site and 100 nucleotides upstream. In addition, to ensure that each PAS site was assigned to the correct gene, this set of PAS sites was refined further by filtering out sites localized within the 3′ UTR lacking reads spanning an upstream exon. To define PAU, we computed the ratio of the number of reads mapping to a PAS site to the number of reads mapping to all PAS sites within the same gene. This resulted in a set of 27,233 PAS sites within 12,280 genes, 22,311 of which had PAUs > 5% (the “Methods” section). Our Iso-seq data analysis pipeline is available online (the “Availability of data and materials” section). We validated that this set of PAS sites was consistent with previously defined PAS site signatures. For example, we observed enrichment of hexameric signal site motifs, such as AATAAA and ATTAAA, ~20–30 nucleotides upstream of the cleavage site [9, 10, 39, 40] (Fig. 2b, one-sided Fisher’s exact test, OR = 2.16, *p* < 2.2e−6, Additional file 1: Fig. S1b) and enrichment of GT-rich sequences ~10–30 nucleotides downstream of the cleavage site [11] (Additional file 1: Fig. S1c, one-sided Fisher’s exact test, OR = 6.03, *p* < 2.2e−6). Finally, we considered the distribution of the filtered set of reads across all genes in the genome and restricted the specific comparative analyses to 2862 genes with a read coverage of ≥ 40 Iso-Seq reads with a polyA tail to obtain a final set of 4446 high confidence PAS sites with PAUs > 5% (Fig. 2c, Additional file 1: Fig. S1a).

To validate the biological utility of the aforementioned pipeline to identify PAS sites from Iso-Seq data, we applied said pipeline to previously published brain and liver Iso-Seq datasets [41] given that most other studies of APA have focused on identifying PAS sites and calculating PAUs to study differential expression of 3′ UTRs across conditions, such as across tissues. Given that read coverage across most genes in brain and liver datasets was poor (Additional file 1: Fig. S2a), we restricted our analysis to 138 genes supported by at least one read that was informative with regard to the location of a PAS in the 3′ UTR in both the brain and liver datasets. We observed that 30% of genes exhibited preferential usage of more distal PAS sites in the brain (at least 500 bp of distance between sites, or 20% for sites at least 1 kb apart) as compared to the liver, for which just 12% of genes used a more distal site (at least 500 bp difference, or 8% for sites at least 1 kb apart, Fig. S2b). This result is consistent with observations made previously that highlight a global lengthening of 3′ UTRs in the brain [13].

### Assessing PAS site features across APA detection methods

We assessed the ability of QAPA [19], DaPars2 [20, 25], TAPAS [21], GETUTR [27], and APATrap [28], 3′-Seq [33] and Iso-Seq to recapitulate known features of PAS sites. In brief, the five computational tools that leverage  RNA-seq work as follows: QAPA extracts 3′ UTRs for all genes from GENCODE. In addition, QAPA incorporates 3′ UTR and PAS site annotation information from GENCODE [42] and the PolyASite database [43], respectively. Alternatively, a user may provide custom PAS site annotations. QAPA will then quantify PAUs by applying Sailfish [44] to resolve RNA-seq reads that map to loci containing multiple transcript isoforms. DaPars2 is a method that identifies PAS sites de novo and quantifies PAU without annotations. DaPars2 first identifies a distal PAS site for every gene based on where the RNA-seq coverage ends. From this, DaPars2 assumes that a single proximal PAS site exists, and it detects this proximal PAS site as an optical fitting point that can best explain a localized dip in read-density. DaPars2 then quantifies the PAU of proximal and distal PAS sites by adding read counts. TAPAS extracts all 3′ UTRs in a gene according to a genome annotation. It then estimates the read coverage of every 3′ UTR, which is given as input to the time-series data Pruned Exact Linear Time (PELT) algorithm [45] to infer change points in a gene where the read coverage increases or decreases the most. TAPAS then filters all change points to define a true set of PAS sites, and PAUs are quantified as previously described [46]. GETUTR extracts reads that map to annotated 3′ UTRs from a reference genome, makes a density function of RNA-seq data using kernel density estimation with a Gaussian kernel, and identifies PAS sites after using techniques that smooth read coverage. APATrap uses a mean squared error model to identify PAS sites. Finally, as mentioned previously, 3'-Seq is a method that enriches for reads covering the 3' end of genes. 

**Fig. 3:**
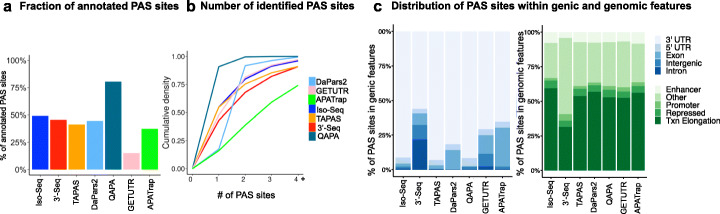
Features of PAS sites. **a** Barplot showing the percentage of PAS sites annotated in the PolyA_DB 3 database [47]. **b** Cumulative density of the number of identified PAS sites identified. **c** Barplot representing the genic location—3′ and 5′ UTRs, introns, exons, and intergenic regions—of PAS sites as defined by HOMER [48] (left), and the genomic locations of PAS sites defined using ChromHMM annotations [49] (right). For the latter, the four annotations represented are enhancer, promoter, repressed, and transcription (txn) elongation. Eleven other annotations were collapsed together as "Other" (see the “Methods” section)

 In total, we ran TAPAS, DaPars2, QAPA, GETUTR, and APATrap using 89 RNA-seq samples from YRI LCLs as input and used our in-house pipelines (the “Methods” section) to process 54 3′-Seq YRI LCL samples and the aforementioned eight Iso-Seq YRI LCL samples. We compared the overall number of PAS sites identified by these five methods and identified 26,545, 22,062, 14,251, 46,169, 12,555, 32,286, and 22,311 PAS sites defined by TAPAS, DaPars2, QAPA, GETUTR, APATrap, 3′-Seq, and Iso-Seq, respectively (PAU > 5%). In addition, we observed that many PAS sites, regardless of which method they were defined by, were previously annotated in the database PolyA_DB 3 [47] (Fig. 3a).

Over Greater than half of the 12,280 genes expressed in LCLs harbor multiple PAS sites, or undergo APA, with 79.8% harboring ≥ 2 PAS sites, as defined by Iso-Seq (Fig. 3a, 9799 genes). Of note, although DaPars2 defines at most two PAS sites per gene, we found that 8% of genes harbored more than two PAS sites (Fig. 3b). This slight discrepancy is due to the fact that we reassigned PAS sites to genes using hg19 RefSeq gene annotations. This was done in order to be able to consistently compare PAS sites across TAPAS, DaPars2, QAPA, GETUTR, APATrap, 3′-Seq, and Iso-Seq in downstream analyses.

Next, we wanted to assess the distribution of PAS sites across genic locations. We observed that while all methods agreed that most PAS sites are localized within 3′ UTRs of genes, 3′-Seq identified a substantial fraction of PAS sites in introns as well (Fig. 3c, 22%). This is consistent with the notion that 3′-Seq is more sensitive to detecting PAS sites  with low PAUs. Indeed, PAS sites in introns were used significantly less frequently than PAS sites in 3′ UTRs (68% and 24% of PAS sites in introns and 3′ UTRs, respectively, with PAUs ≤ 20%, two proportions *Z* test, *χ*^2^ = 4329.5, *p* = 2.2e−16). We also observed greater conservation upstream of PAS sites localized in 3′ UTRs than those in introns or other genic locations, which is consistent with previous findings (Additional file 1: Fig. S3a) [50]. Notably, most PAS sites (range of 31.4–59.3%) defined by all methods were associated with transcription elongation (59.3% of Iso-Seq PAS sites were associated with transcription elongation and 25.1% were associated with other chromatin features, two proportions *Z* test, *χ*^2^ = 6.221, *p* = 6.3e−3), highlighting the importance of local chromatin architecture in PAS site selection (Fig. 3c), as documented previously [51]. Altogether, these results suggest that all methods define PAS sites with at least one established PAS site signature, with Iso-Seq and 3′-Seq identifying an overall greater number of PAS sites with multiple PAS site signatures than the other methods.

### Benchmarking APA detection methods against PacBio Iso-seq to identify PAS sites and quantify PAUs

**Fig. 4:**
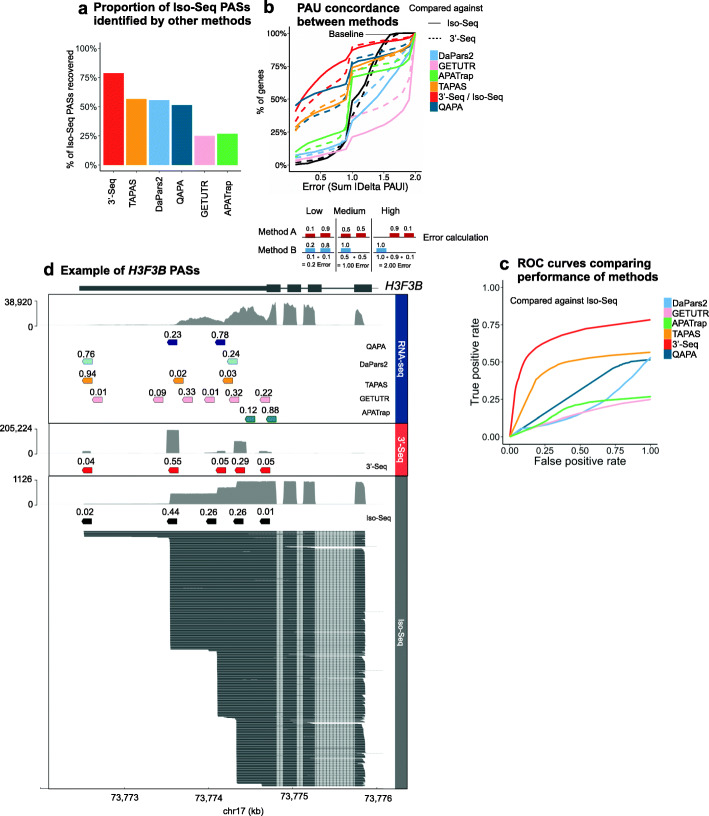
Comparing PAS site identification and PAU quantification across methods. **a** Barplot showing the proportion of Iso-seq PAS sites across 2862 genes that were identified by 3'-Seq and short-read RNA-seq methods. These methods are able to identify, at best, ~75% PAS sites identified by Iso-Seq. **b** Comparison of PAU calls across methods. Error(Sum |Delta PAU|) refers to the concordance in calls between two methods, A and B (as outlined in the schematic). The solid and dotted lines represent comparison of all methods against Iso-Seq, and 3′-Seq, respectively. **c** Receiver operating characteristic (ROC) curves. True positives are instances in which Iso-Seq with PAUs > 5% have analogous PAS sites defined by other methods with PAUs > 0%. False positives are PAS sites defined by other methods with PAUs > 5% that lack analogous PAS sites defined by Iso-Seq. **d** *H3F3B* PAS sites and (PAUs ≥ 1%)

To fairly and directly compare PAS sites defined by TAPAS, DaPars2, QAPA, and 3′-Seq with PAS sites defined by Iso-Seq, we restricted our analyses to the 2862 genes with a read coverage of ≥ 40 Iso-Seq reads. Of the 4446 Iso-Seq PAS sites (PAU > 5%,  ≥ 40 Iso-Seq read coverage), 78.7% were also defined as 3′-Seq PAS sites (Fig. 4a, 3500 PAS sites recovered). In comparison, TAPAS, DaPars2, QAPA, GETUTR, and APATrap were able to recover fewer Iso-Seq PAS sites, at most, 56.6% (Fig. 4a, TAPAS).

To assess if there were observable differences in PAUs between PAS sites within the same gene, we restricted to two PASs within the 3' UTR of every gene within our set of 2862 genes (≥ 40 Iso-Seq reads-- ≥ 40 Iso-Seq reads), the furthest upstream (i.e., proximal) and furthest downstream PAS site (i.e., distal). Interestingly, we observed little difference in proximal and distal PAUs identified by Iso-Seq and 3′-Seq (Fig. S3c). In contrast, TAPAS, DaPars2, QAPA, GETUTR, and APATrap exhibited significant PAU differences between proximal and distal PAS sites (Additional file 1: Fig. S3c, prop.test, *P* = 1.088543e−44). This was expected given that different sequence isoforms can contain a significant amount of sequence overlap, and short RNA-seq reads assigned may be assigned to the incorrect isoform. Therefore, short-read-based methods may overestimate proximal PAU. Indeed, TAPAS and DaPars2 identified a significant number of distal PAS sites with lower PAUs; 74.7% of distal PAS sites identified by TAPAS exhibited PAUs ≤ 50%, and 61.6% of distal PAS sites identified by DaPars2 exhibited PAUs ≤ 50% (Additional file 1: Fig. S3c). Interestingly, QAPA identified slightly more proximal PAS sites with lower PAUs (Additional file 1: Fig. S3c). This may be a result of the fact that QAPA was run with additional annotation information as compared to TAPAS and DaPars2.

Next, we compared PAUs across the different methods at the gene-level. For example, if two methods called the same PAS sites for a specific gene, we computed the difference in their PAUs and took the sum of all the differences across all PAS sites within that gene (Fig. 4b, Additional file 1: Fig. S4b). We defined this as the amount of “error” or difference between PAUs estimated by different methods (Fig. 4b). Examples of low error include cases in which two methods define the same PAS sites per gene but might estimate slightly different PAUs. In contrast, examples of high error include cases in which two methods might define completely different PAS sites per gene. When comparing 3′-Seq, TAPAS, DaPars2, QAPA, GETUTR, and APATrap against Iso-Seq (Fig. 4b, Additional file 1: Fig. S4b “Concordance with Iso-Seq”), 3′-Seq was most concordant. In contrast, GETUTR was least concordant, with 78.6% of genes tested having an error > 1.0, suggesting a large discrepancy between GETUTR-defined PAUs and Iso-Seq-defined PAUs. As with Iso-Seq, all standard RNA-seq-based tools, TAPAS, DaPars2, QAPA, GETUTR, and APATrap, were similarly concordant with 3′-Seq (Fig. 4b, Additional file 1: Fig. S4b “Concordance with 3′-Seq”).

We generated a receiver operating characteristic (ROC) curve to highlight the tradeoff between sensitivity and specificity of 3′-Seq, TAPAS, DaPars2, QAPA, GETUTR, and APATrap as compared to Iso-Seq PAS sites, which, for the purpose of this analysis, were considered to be the ground truth (Fig. 4c). We did not simulate synthetic datasets for this analysis as, to date, there are very few methods that can simulate Iso-Seq data, and existing methods simulate reads lacking polyA tails, rendering them uninformative for the study of APA. We defined a true positive as an instance in which a method identifies a PAS site (PAU > 0%) that overlaps with an Iso-Seq PAS site (PAU > 5%). In contrast, we defined a false positive as an instance in which a PAS site is defined by 3′-Seq, TAPAS, QAPA, GETUTR, APATrap, or DaPars2 with a PAU > 5%, but there does not exist an overlapping PAS site defined by Iso-Seq. Overall, 3′-Seq outperformed all other methods, as measured by the area under the curve (AUC) (Fig. 4c, AUC = 0.66). In contrast, the AUCs for TAPAS, QAPA, DaPars2, GETUTR, and APATrap were 0.46, 0.50, 0.20, 0.17, and 0.20, respectively. We repeated this analysis, now considering the 3′-Seq PAS sites to be the ground truth, and observed similar results (Additional file 1: Fig. S4e).

To showcase the complexity of PAS site identification and PAU quantification, we highlight the gene *H3F3B* as an example in which Iso-Seq and 3′-Seq identified five PAS sites (PAUs > 1%) with comparable PAUs (Fig. 4d). In contrast, the standard RNA-seq based methods identified overall fewer PAS sites, on average, with PAUs that did not agree with Iso-Seq and 3′-Seq.

### Evaluating the study of inter-individual variation in PAS site choice using different APA detection methods

We have demonstrated thus far that 3′-Seq PAS site identification and PAU quantification is more reliable than methods that leverage standard, short-read  RNA-seq data. Nevertheless, a considerable number of RNA-seq datasets are publicly available, which can be readily used to study APA. Therefore, we sought to evaluate the possibility of combining short-read RNA-seq with a set of PAS site annotations derived from small quantities of 3′-Seq or Iso-Seq to study variation in APA across samples.

As a possible test-case, we set out to use human population-scale RNA-seq data alongside 3′-Seq and Iso-Seq PAS site annotations to study the impact of genetic variation on PAU. While previous studies have focused on comparing PAU across conditions such as cell types, tissues, or species, we note that studying the impact of genetic variation on PAU is simply another type of comparison of PAUs across conditions. In this case, there are three conditions, each one a possible genotype. Moreover, to date, very few studies have quantified APA in human population samples to detect genetic variants implicated in genome-wide APA variation [25, 52], although this may change in the future.

Given that studying the impact of genetic variation on variation in PAU across diverse human populations necessitates the use of well-powered datasets with samples collected from many individuals, and the fact that many such datasets are based on standard, short-read RNA-seq protocols, we first wanted to compare the ability to accurately call APA quantitative trait loci (apaQTL), which link variations in PAU to genotype, using PAS sites defined by TAPAS, DaPars2, QAPA, GETUTR, and APATrap by benchmarking against apaQTL we identified using 3′-Seq data. We next ascertained whether the reliability and reproducibility of apaQTL called using a RNA-seq-based tool, QAPA, could be improved when fed custom PAS site annotations based on small quantities of specialized datasets, such as 3′-Seq or Iso-Seq.

**Fig. 5:**
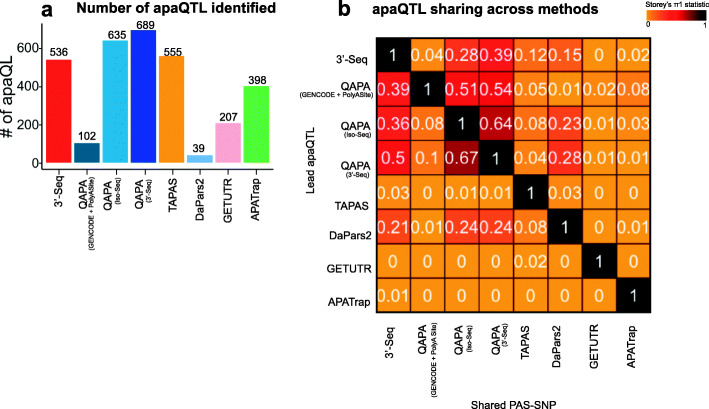
Studying the impact of genetic variation on variation in PAS site choice using different sequencing methods. **a** Barplot showing the number of apaQTL (FDR < 10%) identified across the short-read RNA-Seq-based methods and 3'-Seq. **b** Heatmap showing sharing of apaQTLs across sequencing methods using Storey’s π_1_ statistic. We restricted to apaQTL associated with PAS sites exclusively localized within 3′ UTRs

In order to map apaQTL, we restricted our analysis to samples with genotype information: 87 YRI LCLs for which we had access to short-read RNA-seq data and 51 YRI LCLs for which we had access to 3′-Seq data (the “Methods” section). We did not map apaQTL using Iso-Seq data because data from only 8 LCLs were available to us, much fewer than the tens to hundreds of individuals that are typically required for robust QTL analysis. Briefly, per individual, we defined PAUs for each PAS site defined by every APA method separately, as described previously, now including PAS sites with PAUs ≤ 5%. We quantile normalized these PAUs and tested for association between a PAU and single-nucleotide polymorphisms (SNPs) within 25 kb of the associated PAS site using FastQTL [53]. Significant SNP-PAS site pairs were defined as apaQTL (FDR < 10%). We were able to identify hundreds of apaQTL, but the exact number of apaQTL varied greatly between APA detection methods (Fig. 5a). In particular, we noted a substantial increase in the number of apaQTL called using PAS sites defined by QAPA run with Iso-Seq or 3′-Seq annotations as compared to QAPA run with the default combination of GENCODE and PolyASite annotations (Fig. 5a, 635 and 685 versus 102 apaQTL called by QAPA run with Iso-Seq, 3′-Seq, and GENCODE and PolyASite annotations, respectively). The number of apaQTL called using PAS sites defined by QAPA run with Iso-Seq or 3′-Seq annotations is comparable in number to that of 3′-Seq (Fig. 5a, 635 and 685 versus 536 apaQTL called by QAPA run with Iso-Seq and 3′-Seq annotations, respectively). We observed enrichment of apaQTL near PAS sites, suggesting that all APA detection methods were able to identify many apaQTL that likely enhance or disrupt recognition of signal sites, either directly or indirectly, as expected (Additional file 1: Fig. S5b).

**Fig. 6:**
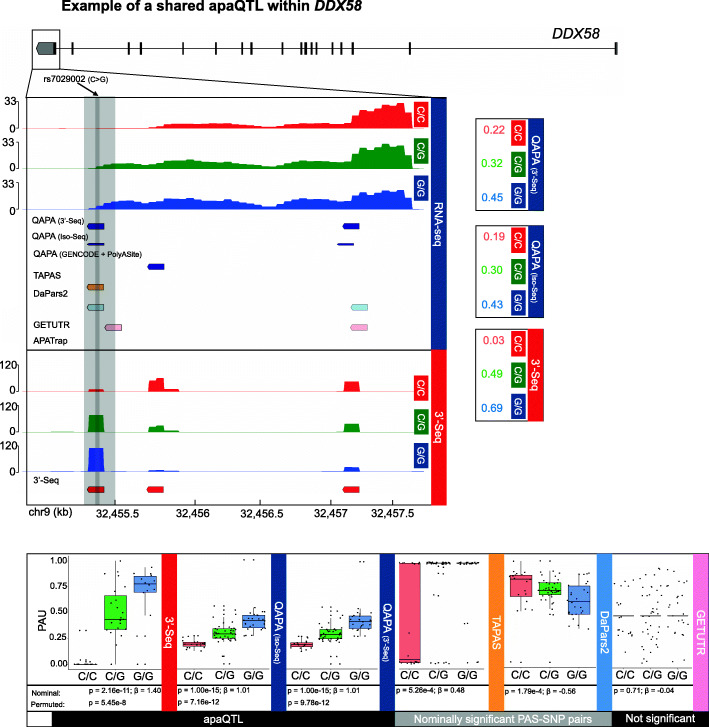
Example of a shared apaQTL in the gene *DDX58* defined by 3′-Seq and shared by QAPA run with Iso-Seq or 3′-Seq PAS site annotations. We highlight a gene track displaying read coverage and PAS sites. The gray vertical track directly below rs7029002 represents the position of the strongest apaQTL SNP, and the surrounding gray track represents the most strongly affected PAS site. PAUs for every PAS site were stratified by genotype, as shown on the right, in which individuals with the G allele have increased PAU of the highlighted, gray PAS site. The bottom highlights boxplots of the PAU at this PAS site, stratified by genotype and APA detection method

To assess what fraction of apaQTL called by the various tools could be recapitulated by other tools, we estimated sharing of apaQTLs using Storey’s *π*_1_ statistic and restricted to PAS-SNP pairs within 3′ UTRs given that some of the short-read RNA-seq-based methods do not identify PAS sites upstream of 3′ UTRs. Interestingly, very few apaQTL called using PAS sites defined by TAPAS, GETUTR, and APATrap were shared with apaQTL called by other methods (Fig. 5b), suggesting potential false positives. We also observed that while only 4% of apaQTL identified by 3′-Seq were shared with those identified by QAPA run with the default GENCODE and the PolyASite database annotations, 28% and 39% of apaQTL were shared by QAPA run with Iso-Seq or 3′-Seq PAS site annotations, respectively (Fig. 5b). For example, rs7029002 (C>G) is an apaQTL identified using 3′-Seq data that was shared with QAPA run with Iso-Seq and 3′-Seq PAS site annotations. As apaQTL represent associations between genotype and PAU, at this locus, individuals with more G alleles at rs7029002 exhibit higher PAUs associated with the PAS site at the end of the 3′ UTR of the *DDX58* gene as compared to individuals with more C alleles (Fig. 6). While this apaQTL is also significant when called using PAUs defined by TAPAS and DaPars2, significance is greatly diminished. Moreover, the estimated effect size is reversed for DaPars2, meaning that in this case, individuals with C alleles, instead of G alleles, exhibit higher PAU of the PAS site at the end of the 3′ UTR (Fig. 6). Interestingly, rs7029002 is upstream of the PAS site it is associated with, suggesting that it is tagging a genetic variant that likely enhances recognition of the cleavage the PAS site, either directly or indirectly. Some apaQTL were exclusively identified by 3′-Seq, such as rs72836634 near the gene *CASC3* (Additional file 1: Fig. S6). Nevertheless, a large fraction of apaQTL called using 3′-Seq could also be identified by running QAPA with Iso-Seq or 3′-Seq annotations (Fig. 5b, *π*_1_ = 0.28 and 0.39), suggesting that running QAPA with Iso-Seq or 3′-Seq PAS site annotations derived from a small number of individuals is a reasonable alternative to exclusively performing 3′-Seq in many individuals for apaQTL mapping.

Lastly, we quantified sharing of apaQTL called using PAS defined by the different tools with expression quantitative trait loci (eQTL), which can serve as a proxy for function. For example, shared apaQTL and eQTL include cases in which one PAS may provide more stabilility to a transcript over another, preventing the transcript from being subject to degradation. In such an example, high gene expression might serve as a proxy for transcript stability. Overall, we observed that sharing of apaQTL with associated gene-SNP pairs was indeed relatively high (Additional file 1: Fig. S5d, *π*_1_ between 0.09 and 0.36). In particular, sharing between eQTL and apaQTL identified using 3′-Seq (*π*_1_ = 0.36) is very similar to that between eQTL and apaQTL identified using QAPA run with 3′-Seq (*π*_1_ = 0.39) or run with Iso-Seq annotations (*π*_1_ = 0.36) (Additional file 1: Fig. S5d). This observation suggests that the apaQTL identified using these three methods are likely to have similar functional impacts on gene regulation.

Overall, these observations suggest that, indeed, under circumstances in which APA-related specialized datasets cannot be generated for a large sample of individuals, QAPA, run with custom PAS site annotations derived from small quantities of such specialized datasets, recapitulates PAS sites and apaQTL that would otherwise be identified using population-scale 3′-Seq data.

## Discussion

Short-read RNA-seq has become central in the assessment of transcriptional and post-transcriptional gene regulatory mechanisms, such as APA, which contributes substantially to the amount of diversity in the human transcriptome and proteome by increasing the number of distinct isoforms produced through differences in PAS site selection. However, given the limited size of RNA-seq sequence fragments and the inherent complexity of the human transcriptome, it remains difficult to accurately reconstruct full-length RNA transcripts with short-read RNA-seq. 3′-Seq, a specialized RNA-seq protocol that enriches for reads at the 3′ ends of genes, is a well-established alternative method used to study APA. Moreover, single-molecule long-read RNA-seq, such as PacBio Iso-Seq, offers a considerable advantage over short-read sequencing to more precisely identify PAS sites and quantify PAUs across mammalian transcriptomes because the protocol allows one capture of full-length transcripts, including polyA tails, thus obviating the need for transcript reconstruction entirely.

In this study, we identified 22,311 PAS sites (PAU > 5%) across 12,280 genes, 38.7% of which are novel, using Iso-Seq data derived from eight LCL samples. We observed that APA detection methods, such as those that take short-read RNA-seq as input, including as TAPAS, DaPars2, QAPA, GETUTR, and APATrap, as well as 3’-Seq, were able to identify comparable numbers of PAS sites. Importantly, PAS sites identified by all methods exhibited well-characterized features of PAS sites, including enrichment of signal site motifs upstream of cleavage sites, enrichment within 3′ UTRs, and association with transcription elongation.

We benchmarked the ability to study APA using with these methods against 3′-Seq and Iso-Seq. We estimated that 78.6% of PAS sites identified by 3′-Seq overlap with Iso-Seq-defined PAS sites whereas, at best, 56.6% PAS sites identified by one of the RNA-seq-based methods overlap with Iso-Seq-defined PAS sites. Moreover, as expected, there is reasonable concordance in the identification of PAS sites and estimation of PAUs between 3′-Seq and Iso-Seq. This is in contrast to the greater discordance between RNA-seq-based methods and Iso-Seq, likely because PAS site identification and PAU quantification among the RNA-seq-based methods can be highly variable. Overall, this suggests that researchers should carefully assess which RNA-seq-based method might serve them best based on the exact biological questions they may be interested in answering. Moreover, 3′-Seq should be the method of choice for studying APA when such data can be generated or are available.

We acknowledge that it is not necessarily practical or cost-effective to generate specialized datasets to study APA, especially given that a plethora of short-read RNA-seq data are already publicly available. Through our analysis of inter-individual variation in APA as a test-case, it is apparent that QAPA, an isoform-based RNA-seq method to study APA, paired with PAS site annotations derived using small quantities of specialized sequencing data, such as 3′-Seq or Iso-Seq, may offer a considerable advantage in studying APA in a cost-effective manner in the near term, until it becomes more accessible and inexpensive to study APA extensively using full-length, long-read sequencing.

## Conclusions

This study demonstrates that current methods to study RNA processing events, such as APA, with short-read RNA-seq data suffer from limitations. However, combining large quantities fo RNA-seq data with small quantities of specialized data, in this case, 3′-Seq or Iso-Seq, strikes  an attractive balance between affordability and accuracy in the study of APA.

## Methods

### Cell culture and RNA sample preparation

We cultured 5 Epstein-Barr Virus transformed lymphoblastoid cell lines (LCLs) at 37°C and 5% CO_2_. These LCLs—GM18501, GM18504, GM19144, GM19239, and GM19153—were derived from the Yoruba (YRI) individuals from the International HapMap Consortium. The Coriell Cat #:Research Resource Identifiers (RRIDs) are GM18501:CVCL_P458, GM18504:CVCL_P460, GM19144:CVCL_P525, GM19239:CVCL_9634, and GM19153:CVCL_P531. These lines were authenticated and tested for mycoplasma contamination. Cell culture and RNA extraction were performed as described previously [52]. In brief, cells were grown in a glutamine depleted RPMI [RPMI 1640 1X from Corning (15–040 CM)] with 15% FBS, 2 mM GlutaMAX (from gibco (35050–061), 100 IU/mL Penicillin, and 100 ug/mL Streptomycin. The lines were passaged 3 times, maintained at 8×10^5^ cells, and grown to a concentration of 1×10^6^ cells per mL before RNA extraction, which was performed as described previously [52]. In brief, cells from each line were spun down and pelleted at 200 g at 500 RPM at 4°C for 2 min, washed with cold phosphate-buffered saline (PBS), and spun down again before aspirating the PBS. RNA was extracted using the miRNeasy kit (Qiagen) according to the manufacturer’s instructions, including the DNase step to remove potentially contaminating genomic DNA.

### Long-read RNA-sequencing data mapping, filtering, and quality control

We processed a total of 8 polyA-selected PacBio Iso-Seq LCL libraries [54] Five SMRT bell libraries were generated for the aforementioned YRI LCLs, GM18501, GM18504, GM19144, GM19239, and GM19153, as per the PacBio Iso-Seq protocol described previously [55]. In brief, cDNA synthesis was performed in triplicate, with each reaction starting with 800–1000 ng of total RNA. The samples were sequenced using 4 SMRTcells. We generated consensus circular sequences (CCS), removed primers, demultiplexed samples, and converted to fastqs [54]. In addition to the 5 YRI LCL samples we generated, we leveraged previously published the Central European (CEU) LCL libraries, GM12878, GM12891, and GM12892 (NCBI SRA SRP036136) [37].

Reads were mapped to the hg19 human reference genome using minimap2 (version 2.2.15) separately for every library [38], using the specific parameters *minimap2 -ax splice -uf -C5 hg19.fa <file>.fastq > <file>.sam*. In order to increase power to call PAS sites, aligned reads from the eight libraries were pooled together.

To identify Iso-Seq reads that capture cleavage and polyadenylation events, we searched for reads that contained stretches of adenosines (i.e., polyA tails). PolyA stretches needed to be located immediately after the 3′ end of the alignment (i.e., starts at the base within the read that does not map to the hg19 reference genome, which is otherwise known as the portion of the read that is “softclipped”). We assessed if the softclipped portion of every read contained a stretch of adenosines. We retained the reads if their softclipped segments were < 20 nucleotides in length and were composed of 95% adenosines. Moreover, if the length of the softclipped segment of a read was ≥ 20 nucleotides, we assessed if the first 20 nucleotides of the softclipped segment was composed of 80% adenosines and if the following 20 nucleotides of the softclipped segment was composed of 95% adenosines and retained these reads as containing a stretch of adenosines.

Next, reads with stretches of adenosines were filtered for internal priming or mispriming using an approach similar to what has been described previously [40]. In brief, we extracted 20 base pairs of the genomic sequence flanking the cleavage site (i.e., 10 nucleotides upstream and 10 nucleotides downstream of the base at which the softclipping segment began) and discarded reads that contained 6 out of 10 adenosines upstream or 6 out of 10 adenosines downstream. We considered this final set of reads as having reliable polyA tails, and therefore, we used this set for downstream analyses.

To ensure the validity of our filtering steps, we verified that the set of final reads showed enrichment of hexameric polyadenylation signals (e.g., AAUAAA), as described previously [12, 39, 40]. In addition, we also verified for enrichment of other sequence elements that are known to play an important role in correct cleavage site recognition, namely a downstream element that contains GU-rich sequences [8, 11, 12].

### Iso-Seq PAS site identification and PAU quantification

Putative PAS sites were defined as the ends of the mapped portion (i.e., the cleavage site) of reads and 100 nucleotides upstream. We then refined the set of reads used to define every putative PAS site by filtering out reads that did not map to annotated 3′ UTRs and that did not also span an upstream exon. We then refined this set of PAS sites by restricting to those in annotated genes using the annotatePeaks.pl script (HOMER v4.11) [48]. This script also annotates with information about the genic location, such as the 5′ or 3′ UTR, intron, and exon of a peak, or in this case a PAS site. We restricted to PAS sites that fell within genes with at least ≥ 40 Iso-Seq reads with a polyA tail. PAUs were quantified by counting the number of reads that ended at a particular PAS site divided by the total number of reads that ended at any PAS site within the same gene. For downstream analyses, we restricted to PAU > 5% (Additional file 2).

### 3′-Seq PAS site identification and PAU quantification

We used 3′-Seq data that were generated from 54 LCLs previously (NCBI SRA SRP223759, total fraction) [52]. Reads were aligned to the hg19 reference genome using STAR v2.6 [56]. Next, reads were filtered for internal priming or mispriming by locating a stretch of 6 adenosines in a 22 nucleotide window surrounding the cleavage site (10 nucleotides upstream and 12 nucleotides downstream), similar to [33]. As was done in for the Iso-seq data, we evaluated enrichment of AAUAAA upstream of the cleavage site. From this final set of reads, peaks were identified as described previously [33]. In brief, peaks were identified by convolving the read coverage with the second derivative of a Gaussian filter such that the lowest convolved read coverage value was defined as the peak center. The peak was then extended 100 nucleotides upstream. Peaks supported by fewer than an average of 5 reads were discarded. This set of peaks was then refined by restricting to those in annotated genes as per the annotatePeaks.pl script from HOMER [48], and PAUs were quantified as described previously for Iso-Seq PAS sites*.* For downstream analyses, we restricted to PAU > 5% (Additional file 3).

### Short-read RNA-sequencing data processing and mapping

Standard, short-read RNA-seq data for 89 LCLs (NA18486, NA18487, NA18488, NA18489, NA18498, NA18499, NA18500, NA18502, NA18505, NA18508, NA18510, NA18511, NA18517, NA18519, NA18520, NA18858, NA18861, NA18867, NA18868, NA18870, NA18873, NA1897, NA18907, NA18908, NA18909, NA18910, NA18912, NA18916, NA18917, NA18923, NA18933, NA18934, NA19093, NA19095, NA19096, NA19098, NA19099, NA19102, NA19107, NA19108, NA19113, NA19114, NA19116, NA19117, NA19118, NA19119, NA19121, NA19129, NA19130, NA19131, NA19137, NA19138, NA19141, NA19143, NA19144, NA19146, NA19147, NA19149, NA19150, NA19152, NA19153, NA19159, NA19160, NA19171, NA19172, NA19175, NA19184, NA19185, NA19189, NA19190, NA19197, NA19198, NA19200, NA19201, NA19204, NA19206, NA19207, NA19209, NA19210, NA19213, NA19214, NA19222, NA19223, NA19225, NA19235, NA19236, NA19247, NA19248, NA19256, and NA19257) were obtained from the GEUVADIS project (EBI ArrayExpress, under the accession E-GEUV-1) [56]. In brief, reads were mapped to the hg19 human reference genome using STARv2.6 [56]. Aligned reads were used as input for three different tools, DaPars2 [26], TAPAS [21], and QAPA [19], that allow for identification of PAS sites from RNA-seq data. Because QAPA is an annotation-based method, we used QAPA’s pre-compiled hg19 annotation library (https://zenodo.org/record/1222196/files/qapa_3utrs.gencode.hg19.tar.gz), which is derived from GENCODE [42] and the PolyASite database [43] together, as was done previously [19]. In addition, we also ran QAPA with two other annotation files, namely the BED files of Iso-Seq PAS sites and 3′-Seq PAS sites that we generated, separately. When running QAPA with these custom annotation files, we extended the 3′ UTRs extracted from the hg19 GENCODE gene prediction annotation tables by 1 kb in order to avoid QAPA not identifying PAS sites that were present in our custom annotation files.

Moreover, all RNA-seq based tools output estimates of PAU separately for every individual. Therefore, we averaged PAU across all individuals for downstream comparisons. The PAS sites were re-annotated with HOMER as described previously, and PAUs for every gene were re-scaled to sum to 1.0 if any PAS sites were omitted because they could not be annotated by HOMER. For downstream analyses, we restricted to PAU > 5% (Additional files 4, 5, 6, 7, 8, 9 and 10).

### Assessing the number of PAS sites within annotated PAS site databases

We used hg19 PAS site annotations derived from PolyA_DB 3 (release 3.2, August 2018) [47] to assess the proportion of PAS sites that were previously annotated.

### Conservation analysis

In the analysis of sequence conservation, we used phyloP scores generated on the 46-way vertebrate alignment, restricting to placental mammals. These were downloaded from the UCSC Genome Browser [57].

### Benchmarking short-read RNA-seq tools and 3′-Seq against Iso-Seq

To assess the concordance between PAS site location and PAU quantification defined by the RNA-seq based tools and 3′-Seq as compared to those defined by Iso-Seq, we restricted to PAS sites that fell within the set of 2862 genes with Iso-Seq read coverage ≥ 40. In addition, we restricted to Iso-Seq PAS sites with PAUs > 5%.

We directly assessed the overlap of PAS sites called by different methods using BEDTools [58]. We defined an error metric, Error(Sum |Delta PAU|), which measures the concordance in PAU calls between two methods, A and B. This measure jointly assesses PAS site localization and PAU quantification concordance. In brief, for every gene, we summed over the differences in PAUs between all PAS sites defined by methods A and B.

### Sensitivity and specificity analysis

ROC curves were generated to assess the sensitivity and specificity of the RNA-seq tools and 3′-Seq in accurately identifying PAS sites. Specifically, the Iso-Seq PAS sites with PAUs > 5% were used as the ground truth. True positives are instances in which Iso-Seq PAS sites with PAUs > 5% have analogous PAS sites defined by other methods with PAUs > 0%. False positives are PAS sites defined by other methods with PAUs > 5%, but lack analogous PAS sites defined by Iso-Seq.

### apaQTL mapping

We mapped apaQTL separately for all methods except Iso-Seq, for which we were lacking power to call QTL given our small sample size of eight individuals. For the RNA-seq methods, we removed two individuals, NA18500 and NA18908, due to low confidence in their annotated identity (a remaining total of 87 individuals). For the 3′-Seq, we removed these same two individuals as well as NA19092 due to lack of genotype information (a remaining total of 51 individuals). We analyzed all PAS sites defined by each of these methods, regardless of PAU.

We standardized all PAU measurements across individuals and then quantile-normalized them to fit a standard normal distribution, as described previously [59, 60]. We used principal components analysis (PCA) to regress out confounders. We regressed out four PCs. To map apaQTL, we ran FastQTL and used all SNPs with MAF ≥ 0.05 within ±25 kb of PAS sites [61]. As input, we used SNPs from GEUVADIS [62, 63]. A *P* value from a standard linear regression was extracted from the FastQTL output for every SNP-PAS pair. In addition, the lead SNP-PAS association for every PAS site was obtained from the 1000 permutations performed by FastQTL [61]. apaQTL were defined as SNPs from this set with FDR < 10%.

### eQTL mapping

We mapped eQTL in a fashion analogous to apaQTL*,* now with the molecular phenotype as gene expression instead of PAU*.* The same set of RNA-seq data from 87 individuals was used. The same set of SNPs, with MAF ≥ 0.05 were used, now within ± 1MB of genes.

### Estimation of QTL sharing

To estimate sharing between apaQTL mapped using PAS site derived from different methods, we used Storey’s π1 method (otherwise known as qvalue()), which considers the *P* value of the lead SNP-PAS pair from method A in method B [64]. Similarly, we also estimated sharing between QTL for the molecular traits APA and gene expression in an analogous fashion in which we considered the *P* value of the association between the lead SNP-PAS pair and gene expression level.

## Supplementary Information

**Additional file 1: Fig. S1.** Iso-Seq data filtering criteria for the study of APA. **Fig. S2.** Differential expression of alternative 3’UTRs between tissues. **Fig. S3.** PAS site genic, genomic, and usage features. **Fig. S4.** PAS site identification and PAU quantification across Iso-seq and short-read methods, including QAPA run with different PAS site annotations. **Fig. S5.** Comparison of apaQTL between APA methods. **Fig. S6.** Example of an apaQTL in the *CASC3* gene defined by 3’-Seq exclusively.

**Additional file 2:** Iso-Seq.PAS.sites. Iso-Seq PAS sites with PAU > 5% (BED format).

**Additional file 3:** 3-Seq.PAS.sites. 3’-Seq PAS sites with PAU > 5% (BED format).

**Additional file 4:** TAPAS.PAS.sites. TAPAS PAS sites with PAU > 5% (BED format).

**Additional file 5:** DaPars2.PAS.sites. DaPars2 PAS sites with PAU > 5% (BED format).

**Additional file 6:** QAPA.GENCODE.PolyASite.PAS.sites. QAPA (run with GENCODE and PolyASite annotations) PAS sites with PAU > 5% (BED format).

**Additional file 7:** QAPA.Iso-Seq.PAS.sites. QAPA (run with Iso-Seq annotations) PAS sites with PAU ≥ 5% (BED format).

**Additional file 8:** QAPA.3-Seq.PAS.sites. QAPA (run with 3’-Seq annotations) PAS sites with PAU ≥ 5% (BED format).

**Additional file 9:** GETUTR.PAS.sites. GETUTR PAS sites with PAU > 5% (BED format).

**Additional file 10:** APATrap.PAS.sites. APATrap PAS sites with PAU > 5% (BED format).

**Additional file 11:** Review history.

## Data Availability

The datasets supporting the conclusions of this article are available: the 89 YRI LCL RNA-seq dataset was generated by the GEUVADIS project and is available in the EBI ArrayExpress repository, E-GEUV-1 [56]. The 54 YRI LCL 3’-Seq dataset was generated by Mittleman et al. 2020 and is available in the NCBI Sequence Read Archive Accession (SRA) repository, SRP223759 (total fraction). The 3 CEU LCL PacBio Iso-Seq dataset was generated by Tilgner et al. 2014 and is available in the NCBI Sequence Read Archive Accession (SRA) repository, SRP036136. The 5 YRI LCL PacBio Iso-Seq dataset was generated in the current study and is available in the NCBI Sequence Read Archive Accession (SRA) repository, PRJNA762669 [65]. All reproducible scripts can be found through Zenodo [54]. A more detailed Iso-Seq analysis pipeline is available on GitHub: https://github.com/ankeetashah/Benchmarking-APA under an MIT license [54]. All other scripts are available upon request.
