# Supplementary material for: Benchmarking sequencing methods and tools that facilitate the study of alternative polyadenylation
Source: Genome Biol. 2021 Oct 14;22:291. doi: 10.1186/s13059-021-02502-z (PMC8518154; doi:10.1186/s13059-021-02502-z)
Supplement: Supplementary file 1 — Additional file 1: Fig. S1. Iso-Seq data filtering criteria for the study of APA. Fig. S2. Differential expression of alternative 3’UTRs between tissues. Fig. S3. PAS site genic, genomic, and usage features. Fig. S4. PAS site identification and PAU quantification across Iso-seq and short-read methods, including QAPA run with different PAS site annotations. Fig. S5. Comparison of apaQTL between APA methods. Fig. S6. Example of an apaQTL in the CASC3 gene defined by 3’-Seq exclusively. [file 13059_2021_2502_MOESM1_ESM.pdf]

Fig. S1

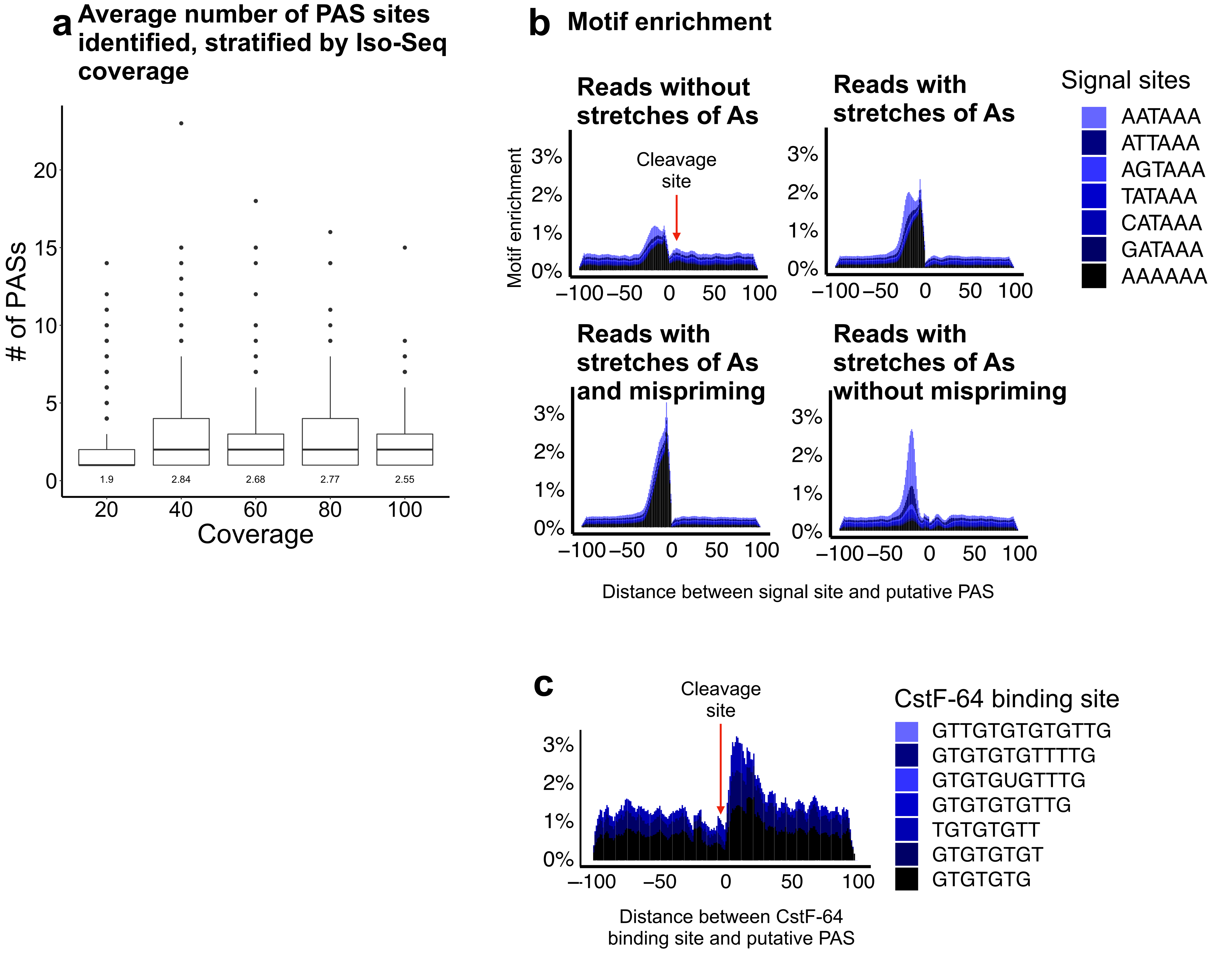

Fig. S1. Iso-Seq data filtering criteria for the study of APA. a Boxplot showing the average number of PAS sites identified per gene, binned by coverage. We observed that genes with coverage ≥ 40 Iso-Seq reads consistently have, on average, 2-3 PAS sites. **b** Meta-gene plots showing enrichment of signal site motifs, AATAAA, ATTAAA, AGTAAA, TATAAA, CATAAA, and GATAAA, 20-30 nucleotides upstream of the putative cleavage site within filtered reads. AAAAAA serves as a negative control. **c** Meta-gene plot showing the enrichment of the GT-rich binding site of CstF-64 10-30 nucleotides downstream of the putative cleavage site.

Fig. S2

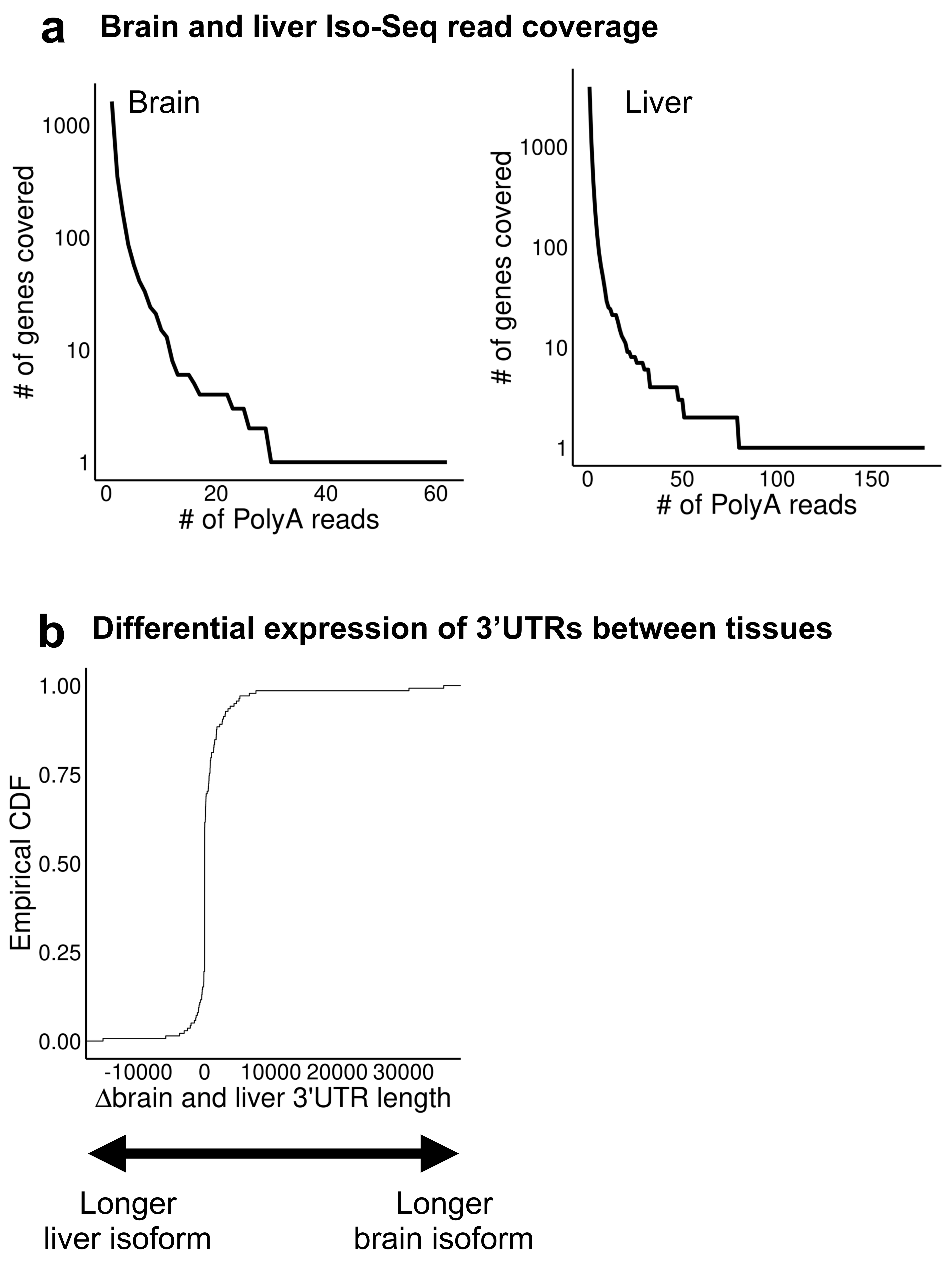

Fig. S2. Differential expression of alternative 3'UTRs between tissues. a Read coverage supporting PAS sites in the 3’UTRs of genes derived from previously published brain and liver Iso-Seq datasets [43] **b** Among 138 genes with a PAS site supported by at least one read, we observed that 30% exhibited use of more distal PAS sites (i.e. longer 3’UTRs, 30% at least 500bp difference, or 20% for sites at least 1kb apart) as compared to liver, which exhibited increased use of more proximal sites (i.e. short 3’UTRs, at least 500kb apart, or 8% for sites at least 1kb apart).

Fig. S3

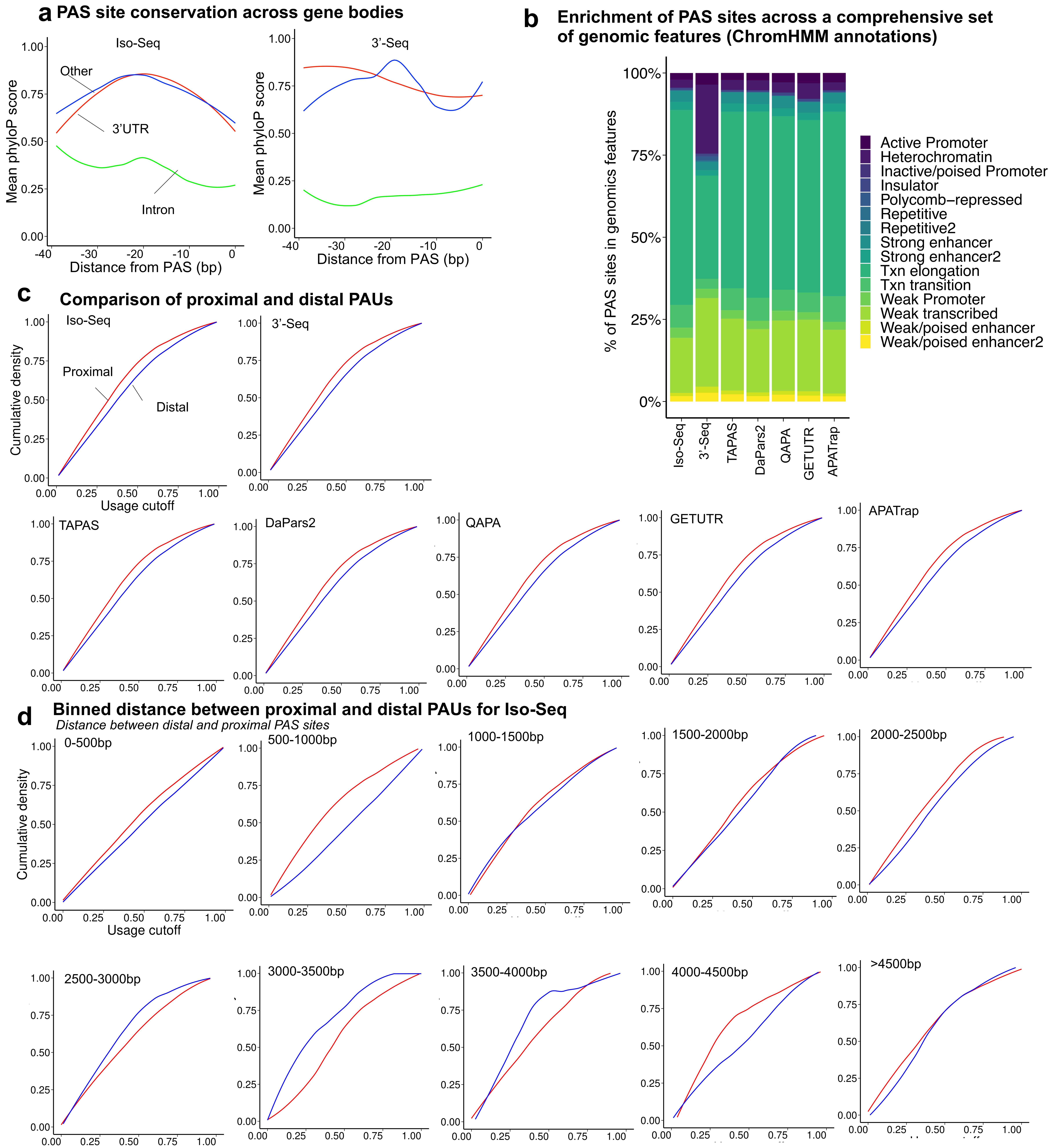

Fig. S3. PAS site genic, genomic, and usage features. a Mean phyloP scores of PAS sites, stratified by localization (*3' UTRs, introns*, or other genic regions labeled as *Other*). PAS sites localized within 3' UTRs tend to be more conserved than those in introns. **b** Barplots showing the genomic locations of PAS sites using 15 ChromHMM annotations [47]. **c** For every gene within our set of 2,862 genes with ≥ 40 Iso-Seq read coverage, we selected PAS sites within 3' UTRs with maximum distance between them and defined the PAS site upstream as the proximal PAS site and the PAS site downstream as the distal PAS site. The proximal and distal PAUs were plotted against varying usage cutoffs. d We binned the distal and proximal PAS sites within 3'UTRs among the 2,862 genes with ≥ 40 Iso-Seq read coverage by distance (500 bp windows). We observed no evidence of PAU estimation bias for short versus long isoforms using Iso-Seq.

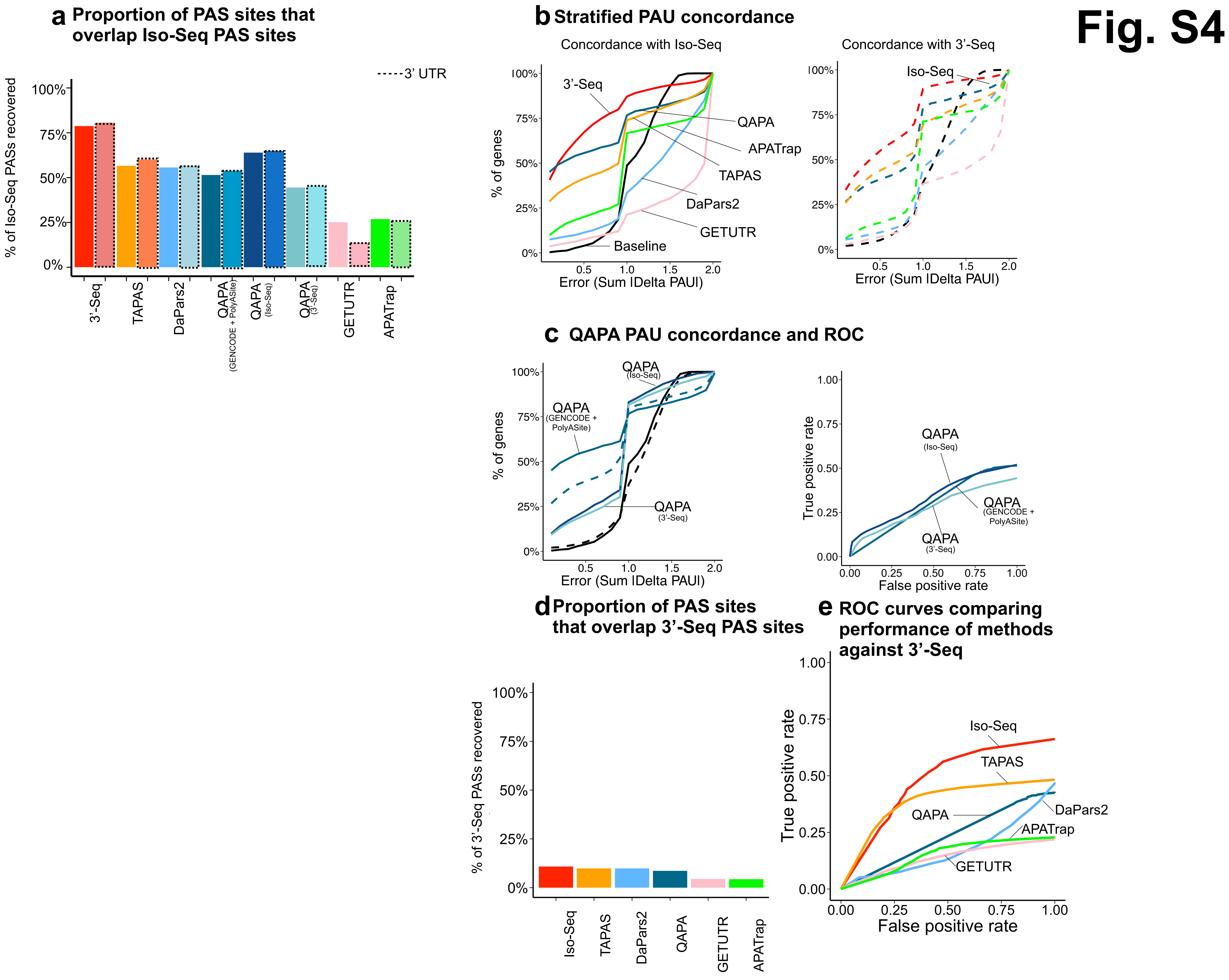

Fig. S4. PAS site identification and PAU quantification across Iso-seq and short-read methods, including QAPA run with different PAS site annotations. a Proportion of PAS sites across 2,862 genes identified by short-read sequencing methods, 3'-Seq, QAPA run with three different PAS site annotations, DaPars2, TAPAS, GETUTR, and APATrap that are also identified by Iso-Seq. Bars with dotted lines represent the % of Iso-Seq PAS sites recovered in 3'UTRs only. **b** Comparison of PAU calls across methods. Error(Sum |Delta PAU|) refers to the concordance in calls between two methods, as per Figure 4. The left compares all methods against Iso-Seq. The right compares all methods against 3'-Seq. **c** Error(Sum |Delta PAU|) receiver operating characteristic (ROC) curve, stratified by distinct QAPA runs using different PAS site annotations, including with GENCODE and PolyASite, Iso-Seq, and 3'-Seq. True positives are instances in which Iso-Seq PAS sites with PAUs > 5% have analogous PAS sites defined by other methods with PAUs > 5%. False positives are PAS sites defined by other methods with PAUs > 5%, but lack analogous PAS sites defined by Iso-Seq with PAUs >5%. **d** Proportion of PAS sites across 2,862 genes identified by Iso-Seq and short-read sequencing methods, QAPA run with three different PAS site annotations, DaPars2, TAPAS, GETUTR, and APATrap that are also identified by 3'-Seq **e** Receiver operating characteristic (ROC) curves. True positives are instances in which 3'-Seq PAS sites with PAUs > 5% have analogous PAS sites defined by other methods with PAUs > 0%. False positives are PAS sites defined by other methods with PAUs > 5%, but lack analogous PAS sites defined by Iso-Seq.

Fig. S5

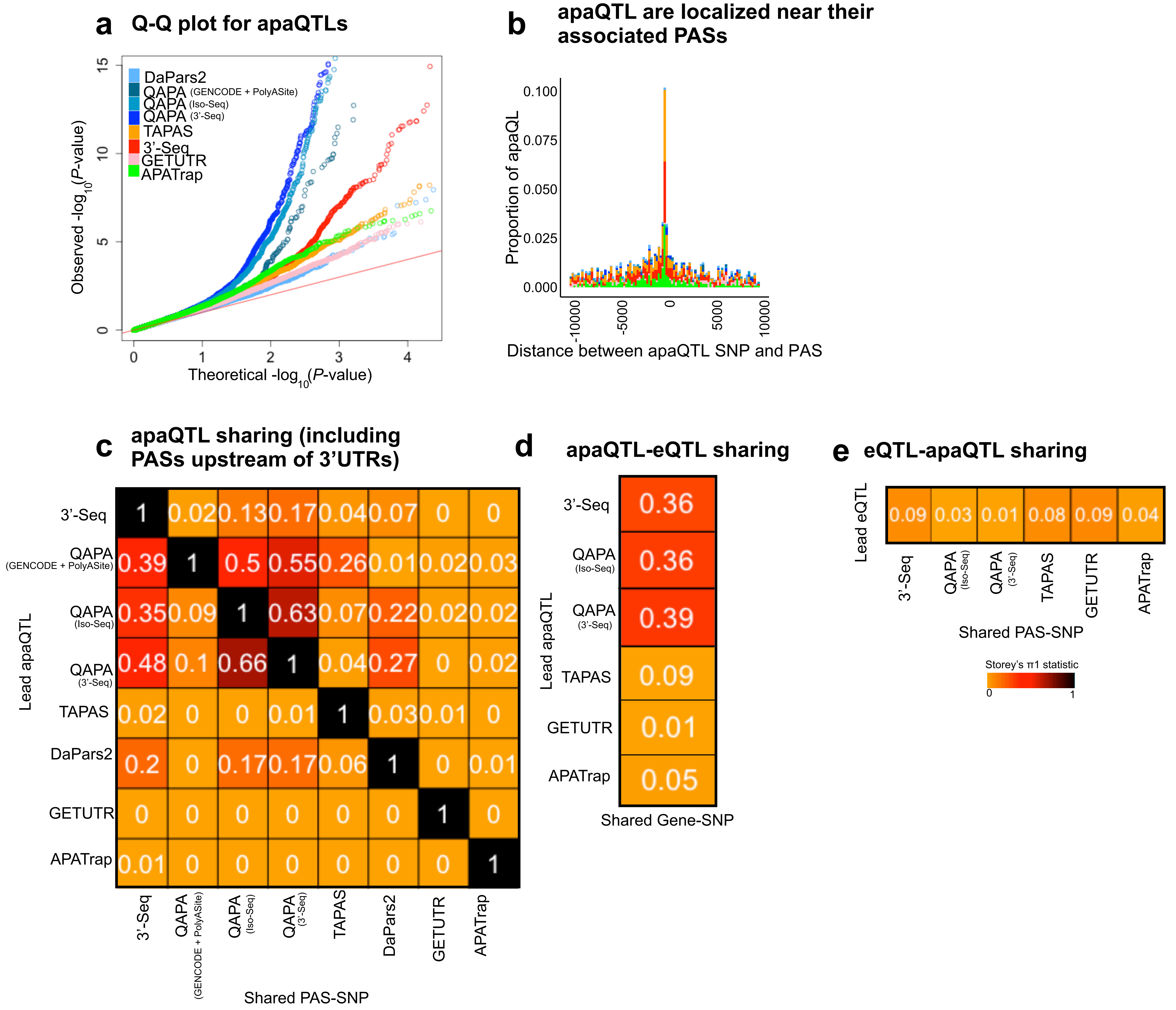

Fig. S5. Comparison of apaQTL between APA methods. a QQ-plot showing apaQTL signals, stratified by method. **b** Location of the lead apaQTL SNP relative to its associated PAS site, stratified by method. **c** Quantification of sharing of the impact of genetic variation on APA across sequencing methods using Storey's π1 statistic. This analysis includes apaQTL linked to PAS sites within and upstream of 3'UTRs. **d** Storey's π1 statistics quantifying the sharing between the lead apaQTL across the methods and most significant Gene-SNP pair. **e** Storey's π1 statistics quantifying the sharing between the lead eQTL and most significant PAS-SNP pair per gene (2,864 eQTL, FDR < 10%).

Fig. S6

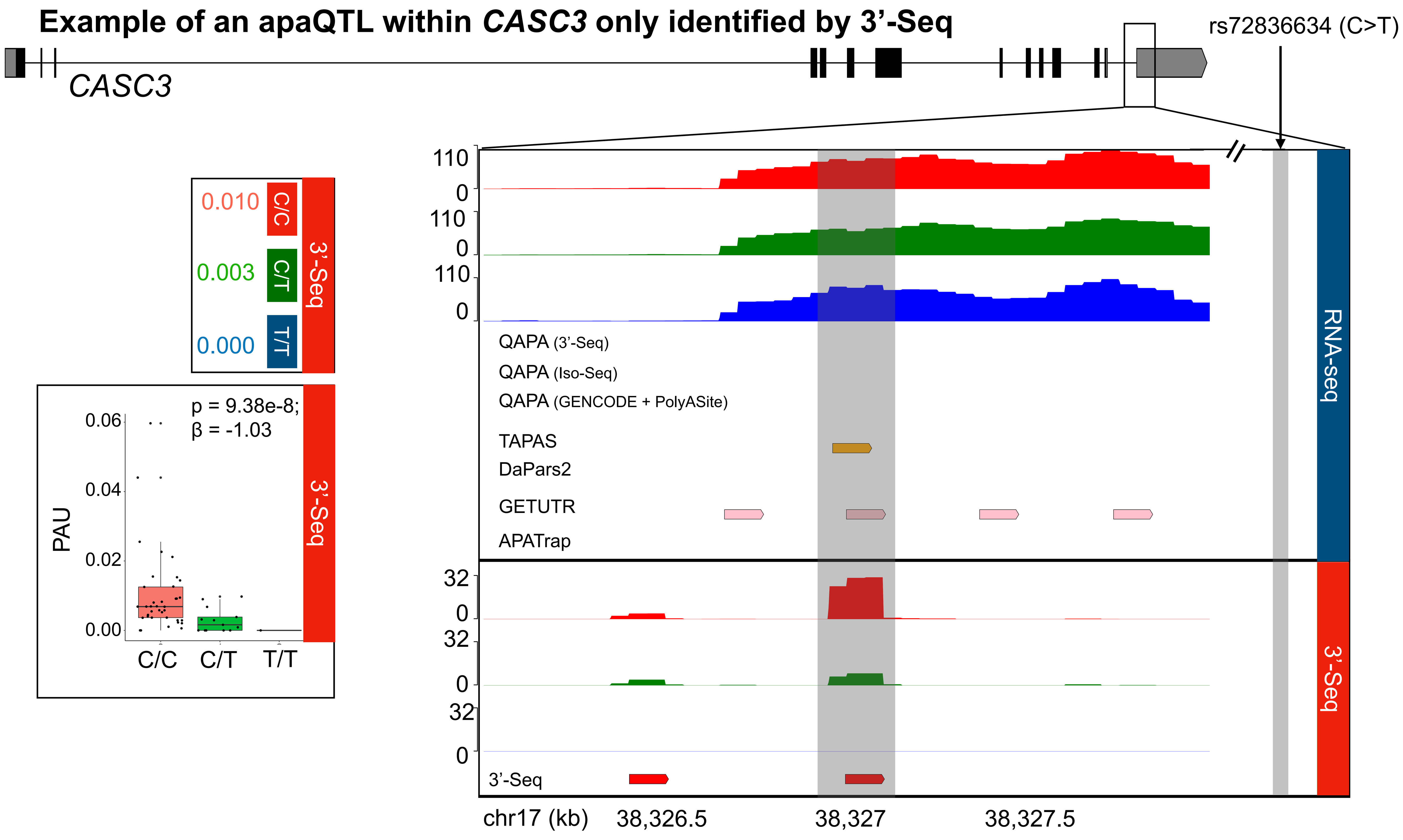

Fig. S6. Example of an apaQTL in the *CASC3* gene defined by 3'-Seq exclusively.
