# Supplementary material for: Benchmarking sequencing methods and tools that facilitate the study of alternative polyadenylation
Source: Genome Biol. 2021 Oct 14;22:291. doi: 10.1186/s13059-021-02502-z (PMC8518154; doi:10.1186/s13059-021-02502-z)
Supplement: Supplementary file 11 — Additional file 11: Review history. [file 13059_2021_2502_MOESM11_ESM.docx]

Review History

**First round of review**

**Reviewer 1**

**Are you able to assess all statistics in the manuscript, including the appropriateness of statistical tests used?**

No, I do not feel adequately qualified to assess the statistics.

**Comments to author:**

The manuscript by Shah et al, describes a timely benchmarking of bioinformatic methods to assess their viability for robust detection of APA in RNA-seq data. Using 3'Seq and Iso-seq (direct RNA sequencing) as controls, the authors show what many labs have likely struggled with, ie that the bioinformatic tools do not work well on their own or with generic APA-database inputs. However, the authors show that there is value in generating a sample-specific 'gold-standard' of polyadenylation positions to guide the bioinformatic detection. Using this approach, they demonstrate that explanatory APA-QTL can be recovered from RNA-seq data.

Specific comments.

1. It is unclear why only three of the now many more bioinformatic tools were benchmarked (and cited) some explanation seems necessary.

2. The focus on problems with 3'-seq (short-read/ PCR bias lines 83-) is irritating and pointless given the end-result that shows it does pretty well anyway… Similarly, the focus on Iso-Seq as the 'gold-standard' assumes no-bias in that approach, which is highly unlikely. As written, it is unclear if the same filtering, read-depth etc… was used between the two approaches to make the comparison anyway. For example, in the legend to figure 4; sites that are not identified by Iso-seq are called false positives, that seems a bold claim without 3'RACE to validate it. It is easy to imagine that RNA structure would generate some false negatives in Iso-Seq. Given that the concordance for the measure that matters (changed isoform usage) is 97.7%, it seems wise to just say two approaches were used to determine the 3'-ends, without an added a value judgement.

3. The work with apaQTL is very promising. It would be important to see what proportion of the inter-individual variance in PAUs is explained by genotype. And, how PAU influences gene expression (ie stabilising/destabilising the rest of the transcript).

4. Throughout the manuscript PAS is used interchangeably to indicate the poly(A) signal and the poly(A) site. The convention is to use it for the former.

Comments on Figures:

Figure 1a: 1-isoform, the schematic RNA doesn't explain the density of reads below. Suggest adding a grey dashed line for the putative extension. Note also that the red and blue reads extend beyond the line where the poly(A) would have started. Those lines needed to stop sharply. Same for 3'-seq schematics.

Figure 1b: This is an unfair comparison. If a statement about precision of abundance measure is to be made (as in line 82) then compare like to like ie just show the ~200bp up and down from the poly(A)-position for all samples. Ideally this point would be made with a scatter plot and correlation statistic to be meaningful. Note it is incorrect to call this the TTS given that transcription is generally terminated some ways down-stream from the site of polyadenylation. It is unclear what the numbers on the y-axis represent.

Figure 4b: There is a problem the 3'seq read coverage. It looks like its been clipped or is in log scale, the read-coverage does not make sense with the numbers under the peak. Its curious that both 3'-seq and Iso-Seq show read coverage for a down-stream site that isn't being called. Why is that?

Minor points

Figure 2A in line 112 and Figure 2a in line 118

Line 198: It is misleading to say the majority (makes it sound like a big difference) when in fact its only slightly more that 1/2

Line 302: A possible use-case ? should this be test-case?

Line 355: the sentence starting 'Individuals with a G allele' comes out of the blue, maybe need an explanatory lead-in sentence.

Line 573: This was not in the Ms (?)

**Reviewer 2**

**Are you able to assess all statistics in the manuscript, including the appropriateness of statistical tests used?**

Yes, and I have assessed the statistics in my report.

**Comments to author:**

In the manuscript by Shah et al., entitled, "Benchmarking sequencing methods and tools that facilitate the study of alternative polyadenylation", the authors analyze long-read PacBio sequencing (Iso-seq) libraries to ascertain its usefulness for APA quantification. They benchmark their analysis against short-read RNA-seq libraries and corresponding tools including TAPAS, QAPA and DaPars2. They also incorporate 3'-seq data from same lymphoblastoid cell lines (LCLs) to identify polyadenylation sites (PASs) and quantify polyadenylation site usage (PAU) . While 3'-seq has long been the reliable technique to identify alternative PASs, various computation tools applied to conventional RNA-Seq data can have some utility. In the manuscript, a great amount of data processing and comparisons were well described and performed, appropriate statistics were applied, and results were well presented that generally support the conclusions.

Despite the thorough analysis, the impact of this work in its current form is limited. Both 3'-seq and Iso-seq use 3' polyA tail orientated reverse transcription strategy in library preparation- thus, it is completely expected that the data would provide largely overlapped 3' end information. The main conclusion of this work is that 3'-seq is better suited to identify and quantify alternative polyadenylation (APA) events. But this is already well established. Many different 3'end sequencing methods have been developed to quantify APA, and many have existed for many years now. There is really no debate on whether 3'seq is better than analysis of short read RNA-Seq data for APA analysis. 3'-seq is better than Iso-seq when considering very long distal 3'UTRs (more details in major comment 2).

The Iso-seq reads filtering and the following 3'UTR annotation is novel and of potential interest, but this is not the main goal of this work, and the authors also do not make it a user-friendly pipeline/package for people to test/apply it. To give an analogous example- the original QAPA paper (Ha et al., 2018, Genome Biology) did not devise completely novel computation tools (It uses existing 3' end annotations combined with Sailfish/Salmon to obtain PAUs); but this package, nonetheless, was made publically available in an easy to use format, thus leading to its use in the community. The authors could increase the impact of their work by making their processing pipeline available as a package for Iso-seq data analysis. There was no public deposition of the analysis software in the submitted manuscript for the reviewers to test it.

Major comments:

(1) An important reason people would like to calculate PAUs is that they want to know the differential expression of alternative 3'UTRs across cell types, developmental stages and tissues. The authors should at least show that Iso-seq based data analysis is able to detect APA events like lengthening or shortening in specific cells/tissues before they claim it as the "gold-standard". All the other methods listed in the manuscript have previously been shown to be able detect such changes.

(2) Methodological details are insufficient regarding the data processing for 3' end quantification from iso-seq data.

(3) Full-length sequencing modalities such as PacBio and Nanopore tend to show less coverage over longer isoforms when compared with short isoforms of a given gene, especially when the extended 3'UTR region is very long, (3 kb and longer 3'UTRs are common in mammalian genomes). Using the current criteria as shown in the manuscript (>40 reads), the longest isoforms will easily be filtered out because of the low depth (for instance, the example gene H3F3B shown in Fig 3, this is already a small gene, only ~ 3.6kb, the distal isoform was omitted in Iso-seq analysis while all the other methods were able to identify it). Therefore proximal isoforms are overestimated, given the sequencing bias toward proximal PASs (longer isoforms are filtered out), so it is hard to say that it is comparable to PAUs from other methods. When the authors use cutoffs like PAU>0.05, this could potentially cause even more problems.

Minor comments:

(1)	The authors mention many times in their narrative that Iso-seq is PCR amplification-free and thus can avoid the bias introduced by PCR amplification in short-read library preparation. Normally after first-strand synthesis there is, in fact, cDNA PCR amplification applied in Iso-seq library preparation. This is included for many full-length cDNA synthesis kits, such as NEBNext, Clontech SMARTer, etc.

(2)	Although the authors generated some Iso-seq data (only 3 of 8 Iso-seq data sets used in this paper were newly generated), the authors admit that they can't map apaQTL for Iso-seq because of "lacking power to call QTL given our small size of eight individuals". Also to increase power to call PASs, they have to pool the reads from all 8 data sets. This indicates that simply more data sets are required to obtain sufficient depths to perform the study.

(3)	In Fig 1, the legend says both TAPAS and DaPars2 use a check-point model whereas in the figure only TAPAS is shown as a check-point method.

(4)	Analysis of apaQTL and eQTL is extensive (2 out of 6 main figures and 2 out of 5 supplementary figures), but there is little APA differential expression analysis. It seems to me that a major goal of this work would be to test the utility of 3'UTR identification and quantification tools. The QTL work is a digression that has too much emphasis in the present manuscript.

(5)	QAPA itself does not identify novel PASs, and relies on existing 3'UTR annotation. When comparing QAPA with other tools like TAPAS and DaPars2 for 3'UTR identification, basically the authors are comparing them with current annotations (GENCODE and polyASite). I do not see the point to do so, or at least they should not emphasize that they are comparing QAPA identification vs TAPAS/DaPars2, et al, since this could be interpreted as misleading.

(6)	Schematics in Fig1a are showing splicing instead of 3'UTRs using horizontal lines.

(7)	All the lines representing Iso-seq reads in Fig 1 and Fig 2 are spanning introns without splicing junctions.

(8)	The way the authors show QAPA strategy in Fig1a is incorrect. Not all the reads covering shared 3'UTR region are considered as "from short 3'UTR isoforms" (if blue means short and red means long, which is not specified in the figure legend).

(9)	Pg12 line 223, it is stated that 3'-seq and Iso-seq detected more PASs with different PAS signatures. But this does not mean these PASs are more likely to be real PASs. It actually could mean the exact opposite.

**Authors Response**

**Point-by-point responses to the reviewers’ comments:**

*We wish to first thank both reviewers for their helpful comments. We believe that we were able to address all comments, which helped us substantially improve our manuscript. In particular, we also worked hard to include additional tools for benchmarking as well as to release well-documented code for processing Iso-Seq data to study APA. We hope that these improvements will greatly benefit our scientific community.*

Reviewer reports:

Reviewer #1: The manuscript by Shah et al, describes a timely benchmarking of bioinformatic methods to assess their viability for robust detection of APA in RNA-seq data. Using 3'Seq and Iso-seq (direct RNA sequencing) as controls, the authors show what many labs have likely struggled with, ie that the bioinformatic tools do not work well on their own or with generic APA-database inputs. However, the authors show that there is value in generating a sample-specific 'gold-standard' of polyadenylation positions to guide the bioinformatic detection. Using this approach, they demonstrate that explanatory APA-QTL can be recovered from RNA-seq data.

Specific comments.

1.	It is unclear why only three of the now many more bioinformatic tools were benchmarked (and cited) some explanation seems necessary.

*Thank you for this comment. We initially chose the tools QAPA, TAPAS, and DaPars2 to benchmark by selecting those that were recently published. Moreover, these tools were chosen because they leverage distinct approaches (i.e. isoform versus change-point) and are relatively well cited, and are thus likely used by research groups in the field.*

*There were additional tools that we initially wished to benchmark that are also relatively new, but we were unable to successfully run the associated bioinformatic pipelines and/or packages. For example, we were unable to successfully run PAQR (Gruber et al., Genome Biology, 2018), which is why it was not included in our benchmarking analyses.*

*To address your comment, we have included a sentence in the manuscript that clarifies the motivation behind choosing this specific array of tools: “While there are many computational tools available that allow one to study APA, we chose these tools specifically because they leverage distinct approaches to study APA, e.g. by leveraging annotation databases and estimating PAU based on transcript-level abundance, or by change-point-based detection methods. We aimed to highlight the relative advantages and disadvantages of each method to inform the scientific community about which method may best serve study goals.” In addition, we have now expanded our benchmarking to include two additional tools, GETUTR (Kim et al., Methods, 2015) and APATrap (Ye et al., Bioinformatics, 2018) in all benchmarking analyses performed. These tools leverage change-point or regression models that are distinct from TAPAS and DaPars2. We highlight in the revised manuscript that: “GETUTR extracts reads that map to annotated 3’ UTRs from a reference genome, makes a density function of RNA-seq data using kernel density estimation with a Gaussian kernel, and identifies PAS sites after using techniques that smooth read coverage. Finally, APATrap uses a mean squared error model to identify PAS sites.” Overall, GETUTR and APATrap miss many PAS sites identified by Iso-Seq and 3’-Seq (see Figure 4b).*

2.	The focus on problems with 3'-seq (short-read/ PCR bias lines 83-) is irritating and pointless given the end-result that shows it does pretty well anyway… Similarly, the focus on Iso-Seq as the 'gold-standard' assumes no-bias in that approach, which is highly unlikely. As written, it is unclear if the same filtering, read-depth etc… was used between the two approaches to make the comparison anyway. For example, in the legend to figure 4; sites that are not identified by Iso-seq are called false positives, that seems a bold claim without 3'RACE to validate it. It is easy to imagine that RNA structure would generate some false negatives in Iso-Seq. Given that the concordance for the measure that matters (changed isoform usage) is 97.7%, it seems wise to just say two approaches were used to determine the 3'-ends, without an added a value judgement.

*Thank you for this very important comment. We have removed such lines that reference biases associated with 3’-Seq while retaining lines that outline some of the biases associated with non-specialized or more standard, short-read RNA-seq protocols.*

*We agree that Iso-Seq is likely to also have biases. That being said, we would argue that nevertheless, the Iso-Seq protocol is likely to be of use for studying cleavage and polyadenylation due to the ability of the protocol to sequence through polyA tails and the reads are full-length. We have now carefully gone through our manuscript and updated our language to reflect our belief that Iso-Seq should not be considered the ‘gold-standard.” Rather, we simply emphasize that it may useful for PAS site discovery, and as such, is worth benchmarking against. Moreover, we highlight that 3’-Seq is an excellent alternative, and it does indeed perform very well. For the latter, we have included additional benchmarking, which we believe will help the readers further evaluate the performance of these standard, short-read RNA-seq-based tools against 3’-Seq. For example, we note the addition of the two supplemental figures, Figure S4d and S4e that highlight the proportion of PAS sites that overlap 3’-Seq PAS sites and ROC curves in which we assume that 3’-Seq PAS sites are the ground truth. The performance of methods is similar, regardless of whether comparisons are made against Iso-Seq or 3’-Seq (compare Figure 4c against Figure S4e).*

*We apologize that the filtering that we used was not immediately obvious. To make comparisons in a consistent and fair way, we did restrict all benchmarking analyses (i.e. Figure 4) comparing RNA-seq based tools against Iso-Seq, RNA-seq-based tools against 3’-Seq, and 3’-Seq against Iso-Seq to the same set of 3,548 genes. These are genes that had sufficient read coverage according to Iso-Seq (i.e. ≥ 40 Iso-Seq reads covering the gene). We have updated our language to reflect this: “To make fair and direct compare PAS sites defined by TAPAS, DaPars2, QAPA, and 3’-Seq with PAS sites defined by Iso-Seq, we restricted our analyses to the 3,548 genes with high confidence Iso-Seq PAS sites. Moreover, PAS sites defined by all tools with PAUs < 5% were filtered out.”*

*Finally, we would like to note here that our conclusion is not that the concordance between 3’-Seq and Iso-Seq is incredibly high, but rather than they are most concordant with each other. The 97.7% value represents the percent of genes for which the “error” between Iso-Seq and 3’-Seq is at most 1.0, and a 1.0 error may still be regarded as a significant difference (see Figure 4b, in particular, the schematic). For example, these are cases in which one method may completely miss a PAS site defined by another method or the PAUs defined by the two methods might be very different.*

*We have now updated the text to clarify this position by removing the line that includes this percentage and including an additional line to emphasize what this error term on the x-axis is referring to: “Examples of low error include cases where two methods define the same PAS sites per gene but might estimate PAUs to be slightly different. In contrast, examples of high error include cases where two methods may define completely different PAS sites per gene.”*

3.	The work with apaQTL is very promising. It would be important to see what proportion of the inter-individual variance in PAUs is explained by genotype. And, how PAU influences gene expression (ie stabilising/destabilising the rest of the transcript).

*Thank you for this encouraging comment. We found that the proportion of inter-individual variance in PAUs explained by genotype varies greatly across PAS. This is expected and is consistent with other molecular traits, such as gene expression levels (Gamazon et al., Nature Genetics, 2015,). While we provide a distribution of variance explained below, we have chosen not to add it to our revised manuscript. In this figure, r2 corresponds to the estimated proportion of variance explained using linear regression, between top SNP-PAU pairs, in this case for 3’-Seq.*

*To answer the second question, we found that 36% of apaQTL were also eQTL (as per apaQTL called using 3’-Seq PAS sites as input), suggesting that a substantial proportion of genetic variants that impact APA also impact gene expression (Figures S5d and S5e). We refer the reviewer to our recent paper (Mittleman et al., eLife, 2020) for other interesting findings regarding the connection between apaQTL and molecular phenotypes.*

4.	Throughout the manuscript PAS is used interchangeably to indicate the poly(A) signal and the poly(A) site. The convention is to use it for the former.

*Thank you for this comment. We have now corrected this mistake in our revised manuscript by explicitly defining PAS as poly(A) signal and keeping this consistent throughput the manuscript.*

Comments on Figures:

Figure 1a: 1-isoform, the schematic RNA doesn't explain the density of reads below. Suggest adding a grey dashed line for the putative extension. Note also that the red and blue reads extend beyond the line where the poly(A) would have started. Those lines needed to stop sharply. Same for 3'-seq schematics.

*Thank you for noticing this. We have updated Figure 1a accordingly.*

Figure 1b: This is an unfair comparison. If a statement about precision of abundance measure is to be made (as in line 82) then compare like to like ie just show the ~200bp up and down from the poly(A)-position for all samples. Ideally this point would be made with a scatter plot and correlation statistic to be meaningful. Note it is incorrect to call this the TTS given that transcription is generally terminated some ways down-stream from the site of polyadenylation. It is unclear what the numbers on the y-axis represent.

*We have removed the phrasing in the manuscript referring to precision to avoid confusion: “…allowing for more precise identification of PASs and estimation of isoform abundance.” The goal of Figure 1b was rather to just highlight the general enrichment of reads around a specific gene reference or annotation, such as the transcription termination site, as has been done in other studies, including our own recent study (Mittleman et al., eLife, 2020, Figure 1B, below). We agree with the reviewer that transcription may be terminated downstream of the PAS site, which is why we do see some enrichment of reads downstream of the annotated TTS. The Y-axis refers to read coverage, which we have now included in the figure.*

*Figure 4b: There is a problem the 3'seq read coverage. It looks like its been clipped or is in log scale, the read-coverage does not make sense with the numbers under the peak. Its curious that both 3'-seq and Iso-Seq show read coverage for a down-stream site that isn't being called. Why is that?*

*We apologize for this mistake. We had uploaded a previous version of the figure which removed sites that are used < 5% PAU. We have now updated the figure which we believe addresses both the peak and the clipping.*

Minor points

Figure 2A in line 112 and Figure 2a in line 118

*Thank you. We have changed this to Figure 2a to be consistent throughout the manuscript.*

Line 198: It is misleading to say the majority (makes it sound like a big difference) when in fact its only slightly more that ½

*Thank you. We have changed the wording in the manuscript to now state that “Just over half of the protein-coding and non-coding genes expressed in LCLs harbor….”*

Line 302: A possible use-case ? should this be test-case?

*Yes, this should be test-case. We have updated this in the manuscript.*

Line 355: the sentence starting 'Individuals with a G allele' comes out of the blue, maybe need an explanatory lead-in sentence.

*We have now added a few words to this sentence to make this clearer: “As apaQTL represent associations between genotype and PAU, at this locus, individuals with more G alleles at rs7029002 exhibit higher usage of a PAS site at the end of the 3’UTR of the DDX58 gene as compared to individuals with more C alleles”*

Line 573: This was not in the Ms (?) – discussed in the supplement. What is the second last paragraph before the discussion? “Lastly, we quantified sharing of apaQTL etc” – point to lines at the end

*We performed this sharing analysis to briefly probe, as this reviewer phrased it in a previous comment, how PAU influences gene expression. For example, high sharing of apaQTL and eQTL suggest include cases in which PAS chosen may stabilize a transcript if we observe that gene expression is high or cases in which PAS chosen may destabilize a transcript if we observe that gene expression is low. There are also instances in which apaQTL and eQTL are not shared, which are cases, for example, in which gene expression of a transcript may be altered by factors independent of PAS choice. Given that this is not a major focus of this study, we chose to include the results in the supplement.*

Reviewer #2:

In the manuscript by Shah et al., entitled, "Benchmarking sequencing methods and tools that facilitate the study of alternative polyadenylation", the authors analyze long-read PacBio sequencing (Iso-seq) libraries to ascertain its usefulness for APA quantification. They benchmark their analysis against short-read RNA-seq libraries and corresponding tools including TAPAS, QAPA and DaPars2. They also incorporate 3'-seq data from same lymphoblastoid cell lines (LCLs) to identify polyadenylation sites (PASs) and quantify polyadenylation site usage (PAU) . While 3'-seq has long been the reliable technique to identify alternative PASs, various computation tools applied to conventional RNA-Seq data can have some utility. In the manuscript, a great amount of data processing and comparisons were well described and performed, appropriate statistics were applied, and results were well presented that generally support the conclusions.

Despite the thorough analysis, the impact of this work in its current form is limited. Both 3'-seq and Iso-seq use 3' polyA tail orientated reverse transcription strategy in library preparation- thus, it is completely expected that the data would provide largely overlapped 3' end information. The main conclusion of this work is that 3'-seq is better suited to identify and quantify alternative polyadenylation (APA) events. But this is already well established. Many different 3'end sequencing methods have been developed to quantify APA, and many have existed for many years now. There is really no debate on whether 3'seq is better than analysis of short read RNA-Seq data for APA analysis. 3'-seq is better than Iso-seq when considering very long distal 3'UTRs (more details in major comment 2).

The Iso-seq reads filtering and the following 3'UTR annotation is novel and of potential interest, but this is not the main goal of this work, and the authors also do not make it a user-friendly pipeline/package for people to test/apply it. To give an analogous example- the original QAPA paper (Ha et al., 2018, Genome Biology) did not devise completely novel computation tools (It uses existing 3' end annotations combined with Sailfish/Salmon to obtain PAUs); but this package, nonetheless, was made publically available in an easy to use format, thus leading to its use in the community. The authors could increase the impact of their work by making their processing pipeline available as a package for Iso-seq data analysis. There was no public deposition of the analysis software in the submitted manuscript for the reviewers to test it.

*Thank you for your constructive review. We should note that while we conclude that 3’-Seq is indeed the best method to identify and quantify the usage of PAS, which is, we agree, well established, there are significant differences between PAS location and usage determined by Iso-Seq versus 3’ Seq, which, we believe, is not well known.*

*We believe that another important finding is that the usage and/or location determined by different tools can be highly variable, thus there is room for improvement of such tools and/or there should be considerations to be made regarding which tool to use based on the exact biological question one is interested in answering. We have also revised the manuscript to strengthen this point: “Moreover, as expected, there is reasonable concordance in identification of PAS sites and estimation of PAUs between 3’-Seq and Iso-Seq, with some differences. This is in contrast to the greater discordance between RNA-seq-based tools and Iso-Seq, likely because PAS site identification and PAU estimation among the RNA-seq based tools can be highly variable. Overall, this suggests that one should carefully which RNA-seq-based tools might serve researchers best based on the exact biological questions they may be interested in answering and that 3’-Seq should be the method of choice for studying APA when such data can be generated or are available”.*

*Thank you for your interest in our Iso-Seq pipeline. We have now worked hard to make our pipeline available on GitHub for others to use: https://github.com/ankeetashah/Benchmarking-APA.*

Major comments:

(1)	An important reason people would like to calculate PAUs is that they want to know the differential expression of alternative 3'UTRs across cell types, developmental stages and tissues. The authors should at least show that Iso-seq based data analysis is able to detect APA events like lengthening or shortening in specific cells/tissues before they claim it as the "gold-standard". All the other methods listed in the manuscript have previously been shown to be able detect such changes.

*Thank you for this suggestion. In an attempt to show that Iso-Seq is able to reproduce lengthening of UTRs, we downloaded and analyzed the brain and liver Iso-Seq datasets from Anvar et al., Genome Biology, 2018. Unfortunately, we found that the coverage across most genes was incredibly low (coverage in this case meaning full length reads that did contain polyA tails). Ultimately, only 138 genes had at least a single Iso-Seq read that was informative with regard to the location of a PAS in the 3’UTR in both the brain and liver datasets. We found that of these 138 genes, 30% showed a lengthening of 3’UTRs (at 500bp) in the brain (or 20% at 1kb) compared to liver (12% at 500 bp or 8% at 1kb) showed lengthening in liver, which is consistent with the lengthening in brain more generally that was described in Mayr, Trends Cell Biol, 2017. However, the lack of high quality Iso-Seq data across human tissues prevents us from performing additional, more comprehensive analyses of 3’UTR lengthening or shortening. We have added a paragraph in the revised version of the manuscript describing this analysis:*

*“To validate the biological utility of the aforementioned pipeline to identify PAS sites from Iso-Seq data, we applied said pipeline to previously published brain and liver Iso-Seq datasets [43] given that most other studies of APA have focused on identifying PAS sites and calculating PAUs to study differential expression of 3’ UTRs across conditions, such as across tissues. Although coverage across most genes in brain and liver datasets was poor (Figure S2a), among the 138 genes supported by at least one read that was informative with regard to the location of a PAS in the 3’ UTR in both brain and liver, we observed that 30% exhibited use of more distal PAS sites in brain (at least 500bp of distance between sites, or 20% for sites at least 1kb apart) as compared to liver, for which just 12% of genes used a more distal site (at least 500bp difference, or 8% for sites at least 1kb apart, Figure S2b). This result is consistent with observations made previously that highlight a global lengthening of 3’ UTRs in brain [14].”*

*Figure S2. Differential expression of alternative 3'UTRs between tissues. a Read coverage supporting PAS sites in the 3’UTRs of genes derived from previously published brain and liver Iso-Seq datasets [43] b Among 138 genes with a PAS site supported by at least one read, we observed that 30% exhibited use of more distal PAS sites (i.e. longer 3’UTRs, 30% at least 500bp difference, or 20% for sites at least 1kb apart) as compared to liver, which exhibited increased use of more proximal sites (i.e. short 3’UTRs, at least 500kb apart, or 8% for sites at least 1kb apart).*

(2)	Methodological details are insufficient regarding the data processing for 3' end quantification from iso-seq data.

*We believe this information is included in the Methods under the section entitled “Long-read RNA-sequencing data mapping, filtering, and quality control.”*

(3)	Full-length sequencing modalities such as PacBio and Nanopore tend to show less coverage over longer isoforms when compared with short isoforms of a given gene, especially when the extended 3'UTR region is very long, (3 kb and longer 3'UTRs are common in mammalian genomes). Using the current criteria as shown in the manuscript (>40 reads), the longest isoforms will easily be filtered out because of the low depth (for instance, the example gene H3F3B shown in Fig 3, this is already a small gene, only ~ 3.6kb, the distal isoform was omitted in Iso-seq analysis while all the other methods were able to identify it). Therefore proximal isoforms are overestimated, given the sequencing bias toward proximal PASs (longer isoforms are filtered out), so it is hard to say that it is comparable to PAUs from other methods. When the authors use cutoffs like PAU>0.05, this could potentially cause even more problems.

*Thank you for this comment. While we agree that Iso-Seq likely suffers from biases that would reduce its utility for some cases, we should note that a large fraction of APA occurs between PAS sites that are near each other. Nevertheless, to investigate this potential effect, we looked at the usage of proximal versus distal PAS sites within 3’UTRs as estimated using Iso-Seq (Figure S3d), and binned by the distance between distal and proximal PAS sites (500 bp windows). We observed that, interestingly, proximal PAS sites had lower PAUs than distal PAS sites, regardless of the distance between the proximal and distal PAS sites. This suggests that usage of proximal PAS sites are not overestimated.*

*We apologize for our mistake in Fig 4d. We had uploaded a previous version of the figure which removed sites that are used at < 5% PAU. We have now updated the figure which we believe addresses your comment. We note that the PAU estimated by Iso-Seq (0.02) is very similar to that of 3’-Seq (0.03) (the previous version was clipped).*

Minor comments:

(1)	The authors mention many times in their narrative that Iso-seq is PCR amplification-free and thus can avoid the bias introduced by PCR amplification in short-read library preparation. Normally after first-strand synthesis there is, in fact, cDNA PCR amplification applied in Iso-seq library preparation. This is included for many full-length cDNA synthesis kits, such as NEBNext, Clontech SMARTer, etc.

*Thank you for this comment and catching our error. We have removed mention of the fact that Iso-Seq is PCR amplification-free.*

(2)	Although the authors generated some Iso-seq data (only 3 of 8 Iso-seq data sets used in this paper were newly generated), the authors admit that they can't map apaQTL for Iso-seq because of "lacking power to call QTL given our small size of eight individuals". Also to increase power to call PASs, they have to pool the reads from all 8 data sets. This indicates that simply more data sets are required to obtain sufficient depths to perform the study.

*Thank you for this comment. We generated 5 of the total 8 Iso-Seq datasets used in this paper, but otherwise, yes, this is comment accurate. We have now added the following sentence in the revised manuscript to highlight the fact that a larger number of Iso-Seq datasets would indeed be required to perform apaQTL mapping: “We did not map apaQTL using Iso-Seq data because data from only 8 individuals were available to us, much fewer than the 50 individuals that are typically required for QTL analysis.”*

(3)	In Fig 1, the legend says both TAPAS and DaPars2 use a check-point model whereas in the figure only TAPAS is shown as a check-point method.

*We apologize for this mistake. DaPars2 is indeed a change-point method. We have now updated the figure.*

(4)	Analysis of apaQTL and eQTL is extensive (2 out of 6 main figures and 2 out of 5 supplementary figures), but there is little APA differential expression analysis. It seems to me that a major goal of this work would be to test the utility of 3'UTR identification and quantification tools. The QTL work is a digression that has too much emphasis in the present manuscript.

*Thank you for your comment, but we respectfully disagree that there is too much emphasis on apaQTL. We believe that while most papers on APA deal with the identification of 3’ UTR and PAU across conditions, apaQTL represents a novel avenue of research, which we predict will gain in popularity. Furthermore, comparison of PAU across two conditions is a special case of apaQTL (in which conditions are genotypes, and we are working with 2 instead of 3 genotypes). We therefore think that showcasing the performance of different tools in aiding in the identification of apaQTL is reflective of performance. Moreover, other papers already assess the utility of some of these tools in their ability to detect differential 3’ UTR isoforms across conditions (i.e. tissues). We have now included more in our revised manuscript to highlight this point: “While studies have focused on comparing PAU across conditions, such as cell types, tissues, or species, we note that studying the impact of genetic variation on PAU generalizes comparisons of PAU across conditions. In the case of QTL mapping, there are three conditions, each one a possible genotype. Moreover, to date, only a handful of studies have quantified APA in a human population samples and detected genetic variants implicated in genome-wide APA variation”.*

(5)	QAPA itself does not identify novel PASs, and relies on existing 3'UTR annotation. When comparing QAPA with other tools like TAPAS and DaPars2 for 3'UTR identification, basically the authors are comparing them with current annotations (GENCODE and polyASite). I do not see the point to do so, or at least they should not emphasize that they are comparing QAPA identification vs TAPAS/DaPars2, et al, since this could be interpreted as misleading.

*We agree with you that our comparison can be misleading to some. We have now changed the way we describe this comparison, highlighting that what we previously described to be PAS sites “identified by QAPA” as those that are found in current annotation databases: “Although QAPA is not designed to identify novel PASs, these comparisons highlight the shortcomings of using current PAS site annotation databases.” Our reasoning behind performing this analysis is to evaluate the performance of QAPA using GENCODE/PolyASite annotations, which is the standard way users will run QAPA. Our intention was to highlight that relying on current annotations will overlook many true PAS.*

(6)	Schematics in Fig1a are showing splicing instead of 3'UTRs using horizontal lines.

*We have now updated Fig1a to reflect this.*

(7)	All the lines representing Iso-seq reads in Fig 1 and Fig 2 are spanning introns without splicing junctions.

*Thank you for pointing this out, we have now updated the corresponding figures.*

(8)	The way the authors show QAPA strategy in Fig1a is incorrect. Not all the reads covering shared 3'UTR region are considered as "from short 3'UTR isoforms" (if blue means short and red means long, which is not specified in the figure legend).

*We thank the reviewer for this comment, we have now updated Fig 1a.*

(9)	Pg12 line 223, it is stated that 3'-seq and Iso-seq detected more PASs with different PAS signatures. But this does not mean these PASs are more likely to be real PASs. It actually could mean the exact opposite.

*We have now removed the clause of that sentence stating that these PAS sites are more likely to be real.*

**Second round of review**

**Reviewer 1**

My concerns have been addressed.

**Reviewer 2**

Iso-seq and 3’-seq cloning approaches utilize the same 3’end priming strategy for reverse transcription. Thus, both detect analogous 3’end sequences, but with different lengths. Even without the analysis presented in this paper, the detecting of largely overlapped PAS sites with the two methods is completely expected. Any differences are likely caused by experimental variation and filtering conditions applied in data processing.

As I mentioned in the previous review comments, in my opinion, the most useful part of the manuscript would be to include a user friendly workflow that could be applied to PacBio Iso-seq long reads sequencing data. For such a workflow, the most important and difficult part is filtering reads and making 3’UTR annotations. While I appreciate the effort to make the workflow available on github, it is very rough in its current form and I found many issues regarding the output data (see comments below).

In the revised manuscript the authors did not change the structure of their manuscript and did not make workflow and its test/application a priority. Thus, I think the current version of the manuscript still lacks novelty in general and the pipeline is not in a form that will be widely adopted by other users. In addition, the authors did not make much effort to make the illustrations easy for readers to interpret. In most of the figures, when I examine the details they are quite confusing and are presented in an inappropriate manner (major comment 3 and minor comments).

Major:

1. I did a test run using SRR1163655. After polyA tail fitering (step 6), only ~10% reads retained in noMP.bam file. And I checked the nopolyA file and found many of the reads actually have polyA signal in the softclipped region. Something might be wrong with the script or the filtering requirement doesn’t fit the data well? There is also mispriming of reads after filtering, and sometimes it can make ~ 20% reads from a gene. It seems scanning 10nt upstream for As is not enough. The authors can try even higher standards or change their strategy, such as checking mismatches (flip from G/T/C to A) at the upstream A stretches in those reads.

2. If the authors do think their apaQTL and eQTL analysis are important for this study and will draw broad and intense interest from the APA community, they should at least summarize it in the abstract. Right now there’s not even a single word mentioning it in the abstract. As the authors are not willing to generate more PacBio Iso-seq data to run apaQTL and eQTL, their analysis are restricted to short read RNA-seq, which makes it even less interesting. Due to the lack of data and power to call PASs, less than 20% genes are covered in this study and mostly are quite short, which makes it difficult to test/evaluate their tool in depth. To cover more APA genes and PASs, they could try size selection prior to library preparation.

3. The illustration of sequencing reads is of unacceptable quality, especially when it comes to PacBio long reads. Short reads are in inconsistent formats (color, size, etc), and long reads don’t show splicing very well.

4. From the authors method we can tell that an upstream exon was used to filter the reads (fig.2a and github instruction), but it seems in Fig. 4 that not all the reads are covering the upstream exon or the whole 3’UTR. After going over the whole workflow and major scripts, I believe the exon.bed file for filtering reads covering upstream exons turned out to include the region the whole transcript is flanking, thus the filtering doesn’t have much power. It might be the case that the authors accidentally put the incorrect exon.bed file in the ref directory? Bedtools intersect was designed for traditional short reads data, using it to filter read covering a given region will pick reads skipping the region by mistake.

Minor:

1. line 102 and 114, authors use “some...biases” several times, please address these biases in detail.

2. there’s error in 02_noMP_exon_UTR.py and the instructional commands.

3. in fig4 reads and coverage graph don’t support each other, and they are not aligned to reference (I downloaded the previously published Iso-seq data and mapped the reads to hg19 genome, and am pretty sure the coverage of Iso-seq data shown in the figure doesn’t make sense at all). Short reads coverage also looks odd.

4. there is no scale bar throughout the whole draft.

5. the authors did not provide sufficient information about the way reference 3’UTR and exon files are generated, so currently this workflow cannot be applied to any other organisms.

6. the layout of the main figures (for example fig1, 4, 6) looks messy. Lines cross numbers, borders and bars, reads overlap with each other, etc.

7. in the result section, authors mentioned they did mispriming filtering by “scanning the 10 nucleotides flanking either side of putative cleavage site” (line 152) but in fig2 it appears that they are scanning NNNNN+AAAA. Also the illustration doesn’t show the cleavage site in a proper way.

8. for “python scripts/04_annotate_usage.py -p test -r ref/Refseq2Gene.txt -a “, argument a is missing.

9. commands should be wrapped for each or several steps. Some are missing, like bam to bed conversion. That way authors can test their pipeline in a much easier way to find out the error and also make it more user friendly.

**Authors Response**

**Point-by-point responses to the reviewers’ comments:**

Reviewer #2: Iso-seq and 3’-seq cloning approaches utilize the same 3’end priming strategy for reverse transcription. Thus, both detect analogous 3’end sequences, but with different lengths. Even without the analysis presented in this paper, the detecting of largely overlapped PAS sites with the two methods is completely expected. Any differences are likely caused by experimental variation and filtering conditions applied in data processing.

As I mentioned in the previous review comments, in my opinion, the most useful part of the manuscript would be to include a user friendly workflow that could be applied to PacBio Iso-seq long reads sequencing data. For such a workflow, the most important and difficult part is filtering reads and making 3’UTR annotations. While I appreciate the effort to make the workflow available on github, it is very rough in its current form and I found many issues regarding the output data (see comments below).

In the revised manuscript the authors did not change the structure of their manuscript and did not make workflow and its test/application a priority. Thus, I think the current version of the manuscript still lacks novelty in general and the pipeline is not in a form that will be widely adopted by other users. In addition, the authors did not make much effort to make the illustrations easy for readers to interpret. In most of the figures, when I examine the details they are quite confusing and are presented in an inappropriate manner (major comment 3 and minor comments).

Major:

1. I did a test run using SRR1163655. After polyA tail fitering (step 6), only ~10% reads retained in noMP.bam file. And I checked the nopolyA file and found many of the reads actually have polyA signal in the softclipped region. Something might be wrong with the script or the filtering requirement doesn’t fit the data well? There is also mispriming of reads after filtering, and sometimes it can make ~ 20% reads from a gene. It seems scanning 10nt upstream for As is not enough. The authors can try even higher standards or change their strategy, such as checking mismatches (flip from G/T/C to A) at the upstream A stretches in those reads.

*Thank you for testing our workflow and for pointing this out. We indeed identified an error. We had added the wrong 01_filter_for_polyA.py script version to our GitHub repository: blocks of code missing that caused reads with polyA tails to be added to the nopolyA output file. We have now updated the repository to now include the correct filtering script. We also did a test run on SRR1163655 (*.sort.bam, 714,071 reads, samtools view -F 0x904 -c SRR1163655.sort.bam), and upon applying our filtering script to remove reads without polyA tails and reads associated with mispriming, we were able to recover 376,059 mapped reads in the noMP.bam file (53% retained, through Step 6). We have now also included a test case with the all 8 Iso-Seq libraries pooled together, restricted to chromosome 22, that we hope you and others who may want to run this pipeline may find useful.*

2.	If the authors do think their apaQTL and eQTL analysis are important for this study and will draw broad and intense interest from the APA community, they should at least summarize it in the abstract. Right now there’s not even a single word mentioning it in the abstract. As the authors are not willing to generate more PacBio Iso-seq data to run apaQTL and eQTL, their analysis are restricted to short read RNA-seq, which makes it even less interesting. Due to the lack of data and power to call PASs, less than 20% genes are covered in this study and mostly are quite short, which makes it difficult to test/evaluate their tool in depth. To cover more APA genes and PASs, they could try size selection prior to library preparation.

*Thank you for this comment. We have updated the abstract to highlight the apaQTL analysis:*

*“However, we find that running one such tool, QAPA, with a set of polyadenylation site annotations derived from small quantities of 3’-Seq or Iso-Seq can reliably quantify variation in APA across samples and across genotypes, as demonstrated by the successful mapping of alternative polyadenylation quantitative trait loci (apaQTL).”*

3.	The illustration of sequencing reads is of unacceptable quality, especially when it comes to PacBio long reads. Short reads are in inconsistent formats (color, size, etc), and long reads don’t show splicing very well.

*We have now updated Figure 1a.*

4. From the authors method we can tell that an upstream exon was used to filter the reads (fig.2a and github instruction), but it seems in Fig. 4 that not all the reads are covering the upstream exon or the whole 3’UTR. After going over the whole workflow and major scripts, I believe the exon.bed file for filtering reads covering upstream exons turned out to include the region the whole transcript is flanking, thus the filtering doesn’t have much power. It might be the case that the authors accidentally put the incorrect exon.bed file in the ref directory? Bedtools intersect was designed for traditional short reads data, using it to filter read covering a given region will pick reads skipping the region by mistake.

*Thank you for pointing this out. Indeed, the exon.bed file does include the region containing the whole transcript that is flanking (in other words, the exon.bed contains the 3’ UTR region). We have now filtered out rows that contain 3’ UTR coordinates from the exon.bed file to mitigate this problem. You can find the updated bed files on GitHub.*

*For example, in the H3F3B “case study” that we highlight in Figure 4, now all reads do span some portion of the 3’ UTR and at least the first upstream exon unlike before. The previous version of this figure was generated with the *.noMP.bam that did not have the exon/UTR read filter applied appropriately.*

*Although we did not expect our results to change qualitatively, we re-ran all analyses using the updated *.noMP.bam in which we correctly remove reads that do not flank the upstream exon. We had fewer reads that we passed our filter: specifically, 27,232 PAS sites across 12,280 genes. Upon restricting to genes with > 40 read coverage (2,862 genes, down from 3,548 genes), we were left 7,932 PAS sites. As expected, this slight difference did not result in any qualitative change in terms of comparisons and only resulted in minor quantitative changes.*

Minor:

1. line 102 and 114, authors use “some...biases” several times, please address these

biases in detail.

*Thank you for this comment. We did indeed discuss specific biases earlier in that section:*

*“Specific biases exist due to the fact that standard RNA-seq protocols include multiple PCR amplification steps during library preparation [31]. There is also bias in sequencing repetitive regions [32] and the issue of short reads not aligning uniquely within a reference genome of interest [33].”*

*We updated the end of that section to now specifically outline bias in 3’-Seq:*

*“...overcomes the issue of limited coverage but suffers from some of the other biases associated with standard RNA-seq, such as mapping errors associated with reads derived from repetitive regions in the genome*

2.	there’s error in 02_noMP_exon_UTR.py and the instructional commands.

*We apologize for this.* *There was an indentation error. This has now been corrected.*

3.	in fig4 reads and coverage graph don’t support each other, and they are not aligned to reference (I downloaded the previously published Iso-seq data and mapped the reads to hg19 genome, and am pretty sure the coverage of Iso-seq data shown in the figure doesn’t make sense at all). Short reads coverage also looks odd.

*We believe we address this in your major comment 4. We used pyGenomeTracks (https://github.com/deeptools/pyGenomeTracks) to plot these data (bigwig or bam files), so we are not sure about what exactly you mean by your comments regarding short read coverage. Perhaps it was the default bigwig resolution (which is not 1-bp, but 50bp) that makes the coverage look odd? We have now updated the bigwig resolution to be 1bp.*

4.	there is no scale bar throughout the whole draft.

*All main figures have been updated to include tick marks.*

5.	the authors did not provide sufficient information about the way reference 3’UTR and exon files are generated, so currently this workflow cannot be applied to any other organisms.

*Thank you for this comment. We have now included more information on GitHub (under Step 7: Define PAS Sites) about how we extracted 3’ UTR and exon reference files from the UCSC Table Browser such that others will be able to generate analogous files for other organisms or genome builds.*

6.	the layout of the main figures (for example fig1, 4, 6) looks messy. Lines cross numbers, borders and bars, reads overlap with each other, etc.

*We have made keys and removed lines overlapping numbers so that the main figures are less crowded/easier to read.*

7. in the result section, authors mentioned they did mispriming filtering by “scanning the 10 nucleotides flanking either side of putative cleavage site” (line 152) but in fig2 it appears that they are scanning NNNNN+AAAA. Also the illustration doesn’t show the cleavage site in a proper way.

*The figure was just for schematic purposes. We do indeed scan 10 nucleotides flanking either side of the putative cleavage site, not 5 and 4 adjacent as is shown in the figure. This change has now been reflected in the figure.*

8.	for “python scripts/04_annotate_usage.py -p test -r ref/Refseq2Gene.txt -a “, argument a is missing.

*This has been corrected.*

9.	commands should be wrapped for each or several steps. Some are missing, like bam to bed conversion. That way authors can test their pipeline in a much easier way to find out the error and also make it more user friendly.

*Thank you for this comment. We have updated the pipeline to include all missing steps, such as the bam to bed conversion. We also wrapped steps 6-8 into a bash file in case one would like to run all steps sequentially as a job (for which we also have an example for chromosome 22). However, we have also left the individual steps outlined in the README just in case one would like to make modifications (i.e. work with different reference files, genome builds, etc...).*
